# How to perform prespecified subgroup analyses when using propensity score methods in the case of imbalanced subgroups

**DOI:** 10.1186/s12874-023-02071-8

**Published:** 2023-10-31

**Authors:** Florian Chatelet, Benjamin Verillaud, Sylvie Chevret

**Affiliations:** 1 ECSTRRA Team, INSERM U1153, Université Paris Cité, 1 avenue Claude Vellefaux, 75010 Paris, France; 2 ENT and head and neck surgery department, Lariboisiere hospital, 2 rue Ambroise Paré, 75010 Paris, France; 3 INSERM U1141 “NeuroDiderot”, Université Paris Cité, 75010 Paris, France

**Keywords:** Propensity score, Subset analyses, Interaction, Simulation study

## Abstract

**Background:**

Looking for treatment-by-subset interaction on a right-censored outcome based on observational data using propensity-score (PS) modeling is of interest. However, there are still issues regarding its implementation, notably when the subsets are very imbalanced in terms of prognostic features and treatment prevalence.

**Methods:**

We conducted a simulation study to compare two main PS estimation strategies, performed either once on the whole sample (“across subset”) or in each subset separately (“within subsets”). Several PS models and estimands are also investigated. We then illustrated those approaches on the motivating example, namely, evaluating the benefits of facial nerve resection in patients with parotid cancer in contact with the nerve, according to pretreatment facial palsy.

**Results:**

Our simulation study demonstrated that both strategies provide close results in terms of bias and variance of the estimated treatment effect, with a slight advantage for the “across subsets” strategy in very small samples, provided that interaction terms between the subset variable and other covariates influencing the choice of treatment are incorporated. PS matching without replacement resulted in biased estimates and should be avoided in the case of very imbalanced subsets.

**Conclusions:**

When assessing heterogeneity in the treatment effect in small samples, the “across subsets” strategy of PS estimation is preferred. Then, either a PS matching with replacement or a weighting method must be used to estimate the average treatment effect in the treated or in the overlap population. In contrast, PS matching without replacement should be avoided in this setting.

**Supplementary Information:**

The online version contains supplementary material available at 10.1186/s12874-023-02071-8.

## Background

Randomized controlled trials remain the gold standard for evaluating treatment effects. However, there are several situations where they are challenging to conduct for technical, ethical, or feasibility reasons [1]. This challenge is particularly evident in the surgical field, where comparative studies, often complex to design, face difficulties in inclusion, with patients and surgeons reluctant to randomize because of a strong prior belief in the superiority of one treatment over another [2]. Such a difficulty of randomization is similarly observed when evaluating drug effects in rare diseases such in oncology or in vulnerable populations - such as pregnant women, fetuses, neonates, children, prisoners, persons with physical handicaps or mental disabilities, and disadvantaged persons (“the Belmont report”) [3].

Thus, in these fields, observational studies are frequently used. However, they are subject to many sources of bias because the baseline characteristics of patients receiving the different therapeutic modalities may differ widely regarding important prognostic factors, illustrating the confounding-by-indication bias from nonrandom treatment allocation. These biases should be properly addressed to avoid biasing the treatment estimate [4]. Multivariable regression has been widely used to that end. However, it is at risk of overfitting in the case of insufficient observations relative to the number of covariates. To overcome these limitations, in 1983, Rosenbaum and Rubin proposed the use of a propensity score (PS), corresponding to the individual probability of receiving the treatment as a function of the measured confounders [5]. Samples are matched or weighted [6] to minimize the discrepancies in observed confounders between treatment groups; in other words, individuals are assigned “balancing” weights, derived from their PS, to under- or overrepresent the characteristics of their treatment group compared to the other group. Under the assumptions of consistency, exchangeability, positivity, no interference, and correct model specification, causal estimates of treatment effect can be provided [7]. Although other causal inference approaches such as g-computation, targeted maximum likelihood estimation, and/or a doubly robust estimator may outperform the propensity score-based approaches [8], the propensity score-based approaches are still the most popular ones in the medical literature. This is even more prominent in the surgical setting, where 83.8% of such studies have been reported to use PS matching [9].

Whichever the setting, clinicians and surgeons often have a strong belief regarding which subset of patients may benefit from which treatment. We considered the question of facial nerve resection in patients with parotid cancer as an illustrative example. Facial function weakness is often used as a surrogate of facial nerve involvement, resulting in the choice of nerve resection [10, 11]. However, Park et al. recently demonstrated that approximately 1/3 of patients with preoperative facial weakness do not exhibit any perineural invasion on final pathologic examination [12], so facial nerve sparing could be considered even in this situation. Moreover, facial nerve sacrifice has been reported to significantly reduce the quality of life, despite facial nerve reconstruction [13]. Thus, whether the facial nerve should be resected in all patients with parotid tumors abutting the facial nerve or only in those with facial palsy is a matter of debate.

From a statistical point of view, this issue raises the concern of treatment-by-subset interaction when using propensity score approaches (where, in the example above facial nerve resection and no resection are the two “treatment” groups, and facial palsy or no facial palsy are the two “subsets”). One issue is whether the PS estimation should be performed once for the whole sample before performing any subset analyses (“across subsets” strategy) or within each subset separately (“within subsets” strategy). Indeed, while in theory, the true PS balances the distribution of covariates between subsets, in practice, this action occurs only with a large number of patients (reported above 1,000) and events [14]. Thus, the balance between covariates could be improved by estimating the PS in each subset, although this approach may increase the variance in the estimate, with potential numerical issues if there are few patients in one subset [15]. Otherwise, there are uncertainties concerning the extrapolation of these results when the subsets are very unbalanced, and few studies have considered right-censored outcomes [16].

To address these issues of estimating the PS before assessing treatment-by-subset interactions on a right-censored outcome on observational data, we conducted a simulation study for the case when the subsets are very imbalanced in terms of prognostic features and treatment prevalence. We then illustrated those approaches on the motivating example.

## Motivating example

To illustrate the problem, we used data from an observational prospective multicenter cohort of a French national network, focusing on rare head and neck cancers, the *Réseau d’expertise français sur les cancers ORL rares* (REFCOR) database. Patients were included between 2009 and 2021 at the time of diagnosis and then followed prospectively. Inclusion was carried out by each center using a standardized questionnaire. In accordance with French law, their data were anonymized, and all patients signed an informed consent form.

Only patients diagnosed with a primary histologically proven parotid cancer who were surgically treated and included in the REFCOR database were included. To address the objectives of this work, we selected patients with a tumor that was in close contact with the nerve. Surgical reports were reviewed to assess the relationships between the facial nerve and the tumor, and close contact was defined as a contact with at least one of the following three criteria:

1. strong adhesion with the nerve described by the surgeon
2. peri-neural invasion described by the pathologist
3. inframillimeter surgical margin as defined by the pathologist.

Patients with a metastasis located in the parotid gland, patients without any follow-up data, and patients treated for recurrence were excluded. To resume the prognosis of each patient, we used a validated prognostic score for parotid cancers, developed by Vander Poorten et al. [17, 18], classifying patients into 4 groups representing increased risk of poor survival.

Surgical treatment was performed according to local recommendations after discussion in a multidisciplinary tumor board meeting. For the current study, the treatment of interest was facial nerve resection, defined as resection of the facial nerve trunk or one of its main divisions, for carcinologic purposes.

The primary outcome was overall survival (OS), defined as the time from surgery to death or the last visit. The secondary outcome was disease-free survival (DFS), defined as the time from surgery to death or recurrence (local, regional, or distant) or to the last visit. Survival times longer than 5 years were right-censored.

A total of 707 patients from 21 centers were included in this study (see flow chart in Supplementary Fig. 1, Additional file 1). Among these patients, 300 had a tumor in contact with the nerve, including 178 who benefited from a facial nerve resection. Comparison of these 178 patients with facial nerve resection with those 122 patients who did not have any nerve resection revealed marked differences across groups in key prognostic factors, as measured by standardized mean differences (SMDs), of which all but one were above 10% (Table 1). Patients who underwent facial nerve resection had deleterious outcomes in terms of both OS and DFS (see Additional file 4). Two hundred (66.7%) patients with no FN paresis, compared to 87 (29%) who had pretreatment facial weakness, differed from most prognostic factors, with SMDs above 20% (Table 2). Therefore, estimating treatment-by-subset interaction required a propensity score approach to correct for such a potential confounding by indication bias.

**Table 1:** Baseline characteristics of the population according to facial nerve resection

REFCOR cohort:	No resection	FN resection	SMD
n	122	178	
Pretreatment facial palsy (%)	10 ( 8.3)	77 (46.4)	0.946
Age at diagnosis (mean (SD))	57.18 (18.65)	64.16 (14.05)	0.423
Male sex (%)	67 (54.9)	114 (64.0)	0.187
Tumoral size (mm)	28.34 (18.02)	32.34 (15.56)	0.238
Log(Tumoral size)	3.17 (0.65)	3.35 (0.55)	0.311
Extraparenchymal extension (%)	38 (32.8)	78 (55.3)	0.467
Skin or bone invasion (%)	10 ( 8.6)	20 (14.2)	0.176
cN+ (%)	34 (27.9)	68 (38.2)	0.221
M1 (%)	5 ( 4.1)	15 ( 8.4)	0.179
Grade (%)			0.676
I	44 (38.6)	24 (14.1)	
II	19 (16.7)	18 (10.6)	
III	51 (44.7)	128 (75.3)	
Deep lobe tumor (%)	35 (28.7)	47 (28.1)	0.012
Adenoid cystic carcinoma (%)	18 (14.8)	19 (10.7)	0.123
Total parotidectomy (%)	101 (82.8)	166 (94.3)	0.368
Neck dissection (%)	88 (74.6)	161 (94.2)	0.560
Radiotherapy (%)	86 (72.3)	150 (85.2)	0.321
Chemotherapy (%)	16 (13.6)	48 (27.9)	0.360
Surgical margin (%)			0.236
Negative	23 (20.0)	43 (26.1)	
Positive	63 (54.8)	95 (57.6)	
Close	29 (25.2)	27 (16.4)	
Vander Poorten score (mean (SD))	5.15 (1.17)	5.98 (1.01)	0.763
Prognostic index (%)			0.859
1	13 (15.7)	2 ( 1.9)	
2	26 (31.3)	13 (12.1)	
3	20 (24.1)	26 (24.3)	
4	24 (28.9)	66 (61.7)	

*FN* Facial nerve, *SMD* Standardized mean difference, *SD* Standard deviation, *cN+* Clinically involved lymph nodes, *M1* Metastasis

**Table 2:** Characteristics of the population according to pretreatment facial nerve function

	Normal facial function	Pretreatment facial palsy	SMD
n	200	87	
Facial nerve resection (%)	89 (44.5)	77 (88.5)	1.054
Age at diagnosis (mean (SD))	60.21 (17.68)	64.34 (13.11)	0.266
Male sex (%)	112 (56.0)	60 (69.0)	0.270
Tumoral size (mm)	29.95 (17.10)	31.97 (15.31)	0.124
Log(Tumoral size)	3.24 (0.64)	3.36 (0.48)	0.219
Extraparenchymal extension (%)	78 (40.0)	33 (62.3)	0.457
Skin or bone invasion (%)	17 ( 8.7)	11 (20.8)	0.345
cN+ (%)	58 (29.0)	37 (42.5)	0.285
M1 (%)	9 ( 4.5)	11 (12.6)	0.294
Grade (%)			0.725
I	60 (31.4)	5 ( 6.2)	
II	28 (14.7)	9 (11.2)	
III	103 (53.9)	66 (82.5)	
Deep lobe tumor (%)	53 (26.9)	25 (30.5)	0.079
Adenoid cystic carcinoma (%)	24 (12.0)	11 (12.6)	0.020
Total parotidectomy (%)	177 (88.5)	80 (92.0)	0.117
Neck dissection (%)	163 (83.6)	77 (92.8)	0.287
Radiotherapy (%)	150 (76.9)	76 (87.4)	0.275
Chemotherapy (%)	31 (16.1)	30 (35.7)	0.460
Surgical margin (%)			0.448
Negative	50 (26.7)	14 (17.3)	
Positive	92 (49.2)	57 (70.4)	
Close	45 (24.1)	10 (12.3)	
Vander Poorten score (mean (SD))	5.28 (1.03)	6.66 (0.79)	1.504
Prognostic index (%)			1.342
1	15 (10.3)	0 ( 0.0)	
2	39 (26.9)	0 ( 0.0)	
3	40 (27.6)	6 (14.6)	
4	51 (35.2)	35 (85.4)	

*SMD* Standardized mean difference, *SD* Standard deviation, *cN+* Clinically involved lymph nodes, *M1* Metastasis

## Simulation study

We aim to evaluate from observational data a subset-by-treatment interaction on right-censored data using propensity score methods. To specifically evaluate the empirical performances of the two “across” and “within” subsets strategies, we performed a Monte-Carlo simulation study. We generated data close to the REFCOR setting, where patients with facial palsy, the smaller group of the sample, received mostly (in 80% of cases) a facial nerve resection and may have benefit more to that resection than those without (the majority of the sample but who only received a nerves resection in 40% of cases).

We thus considered a population partitioned into two subsets $(S=1,2)$ of different sizes, with a potential heterogeneity in treatment effect across the subsets. Similarly to the REFCOR study, we considered the treatment effect possibly restricted to the smaller subset, but where the treatment has been widely preferred.

### Data generation-generating mechanisms

We considered a population partitioned into two subsets $S (=1,2)$ of potential differential influence on a right-censored outcome (where large times indicate improved outcomes), with a proportion of $p(S=1)=0.25$ patients in the subset 1 (the smaller subset).

We simulated samples of *n*=3,000 patients, with a set of continuous ($X_1$ and $X_2$) covariates using independent normal distributions of mean 0 and standard deviation of 1, and seven binary ($X_3, \ldots , X_{10}$) covariates using independent Bernoulli distributions, with parameter equal to 0.5. Covariates had a strong, moderate or no association, first with outcome, and second with treatment allocation (Table 3).

**Table 3:** Covariates included in the simulation as a function of their association with treatment allocation and outcome

Outcome	Treatment allocation		
	Absent	Moderate	Strong
Absent	*X*3		*X*1
Moderate	$X9|S=1$	*X*2 ; *X*5	*X*7 ; $X9|S=2$
Strong	$X4|S=2$ ; *X*6	*X*8	$X4|S=1$ ; *X*10

Strong, moderate and absence of impact were set by parameter values of $\log 2$, $\log 1.3$ and $\log 1$, respectively

For subject $i=1,\ldots ,n$, we generated his(her) belonging to subset $S=1$, from a Bernoulli $S_i \sim B(0.25)$ distribution, then generated the treatment group, $Z_{i}$ $\sim B(p_{i})$ with $logit(p_{i})= \beta _{0|S} + \sum _{j=1}^{10}\beta _{j|S}.x_{j,i}$, where $\beta _{0|S=1}$ was set at 0.3 and $\beta _{0|S=2} = -1.9$ to obtain $P(Z_i=1|S=1) \approx 0.8$ and $P(Z_i=1|S=2)\approx 0.4$ (close to the REFCOR proportions of treated patients in each subset); and $\beta _{j|S}$ denote the different covariate effects on treatment allocation.

Each survival outcome $T_{i}$ was then generated an exponential distribution with hazard depending on the treatment $Z_i$, covariates $(x_{ji},j=1,\ldots ,10)$ and subset $S_i$ of the patient, given by $\lambda _{i} = \lambda _0.exp [\theta _S.Z_{i} + \alpha _S.S_i + \sum _{j=1}^{10} \alpha _j.x_{j,i}]$, where the baseline hazard, $\lambda _0$, was set to 0.005, and the conditional treatment effects in subsets 1 and 2 at $\theta _{S=1}$ and $\theta _{S=2}$, respectively, while $\alpha _S$ denote the effect of the subset on the outcome, and $\alpha _j$ denote the covariates effect on the outcome (Table 3). Strong, moderate and no impact were set by parameter values of $\log 2, \log 1.3$ and $\log 1$, respectively.

We simulated an independent censoring time for each patient using a uniform distribution $U=[1,150]$, where patients with a censoring time below the time-to-event, or above 60, were administratively right-censored.

Several scenarios were investigated, depending on the impact of the subsets on the outcome ($\alpha _{S=1}$ and $\alpha _{S=2}$), that is without treatment effect and treatment-by-subset interaction (Table 4). We then assessed the influence of sample size (*n*, from 500 to 5,000), proportions of patients in each subset ($p(S=1))$, relative risks to be treated in each subset ($\beta _{0|S=1}$ and $\beta _{0|S=2}$), and impact of covariates on the outcome ($\alpha _j$).

**Table 4:** Summary of the main scenarios

Scenario	Treatment effect		Interaction
	on the log HR scale		
	Subset 2	Subset 1	treatment-by-subset
	$\alpha _{S=2}$	$\alpha _{S=1}$	
Sc1	0	-0.7	yes
Sc2	0	$\varepsilon$	depending on $\varepsilon$
Sc3	$\varepsilon$	$\varepsilon$	no
Sc4	0	0	no

$\varepsilon$ ranged from 0 down to -2

### Estimand/target of analysis

True causal marginal treatment effect in the treated, as measured on the log HR scale, were computed for each scenario in each subset $S=1,2$, using a sample of 1,000,000 individuals.

### Methods

In looking for treatment-by-subset interaction, two strategies of analysis regarding the PS estimation were considered and applied to each dataset. First, the “across subsets” strategy was used, which consisted of estimating a single PS from the whole sample but incorporating the subset indicator and potential interaction terms into the PS. Second, we applied the “within subsets” strategy, which consisted of estimating the PS in each subset separately.

Regardless of the strategy, several PS methods were applied, targeting the average treatment effect in the treated group (ATT) and then the average treatment effect in the overlap (ATO). Thus, we first performed PS matching without replacement using a 1-to-1 nearest neighbor matching algorithm, with a caliper set to 0.2, then to 0.1 standard deviations of the logit of the PS [19]. The hazard ratio (HR) of an event was then estimated from a Cox model with a robust estimator of the variance. In a second approach, we allowed replacement in the untreated group, calculating the variance in the estimator based on the Austin and Caufri estimator [20]. We also used a PS weighting approach, with standardized mortality ratio weights (SMRWs) [21] after stabilization [22] and with a bootstraped variance estimation [23], and then with overlap weights [24], with a robust estimation of the standard errors to account for weighting.

To evaluate the influence of the PS model, we used different models for PS estimation. “True PS” was defined as multivariable logistic regression including true confounders (variables affecting both treatment allocation and outcome) and interaction terms between the subset and variables with different effects on treatment allocation (*X*4 and *X*9). We further included *X*6 for the “PS with a prognostic variable”, *X*1 for the “PS with an instrumental variable”, interaction terms with *X*2, *X*5, *X*7 and *X*10 for the “PS with all interaction terms”, and we omitted all interaction terms for the “PS without interaction term”.

### Performance measures

To assess the performances of these methods, $n_{sim}=$ 1,000 independent replications of each scenario were performed, corresponding to a $< 1\%$ Monte Carlo standard error, for a coverage of 95% [25].

Over those replications, we computed the mean bias, defined as the average difference between the estimated treatment effect and the true marginal treatment effect, and the coverage of the 95% confidence interval, defined as the percent of time the true treatment effect was included in the 95% confidence interval. We also reported the 95% confidence interval of the bias estimation, using the Monte Carlo standard error, and recorded the frequency of non-convergence issues.

The simulation study and analyses for the applied example were performed in R version 4.1.3 using the “survival”, “survey”, “simsurv”, “ggplot2”, “survminer”, “tableone”, “mice”, “MatchIt”, “MatchThem”, “WeightIt”, “cobalt”, “boot”, “VIM” and “forestplot” packages.

### Results

**Fig. 1:**
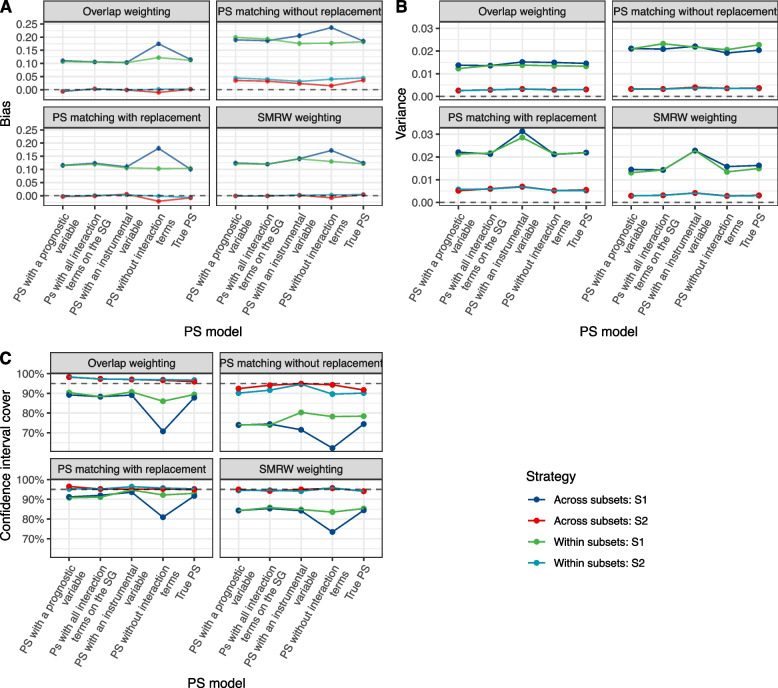
Comparison of strategies according to the PS model in Scenario 1. Comparison of “across subsets” or “within subsets” strategy in terms of the mean absolute bias (**A**), variance (**B**) and the coverage of the 95% CI (**C**) according to the PS model. S1 = subset 1; S2 = subset 2

**Fig. 2:**
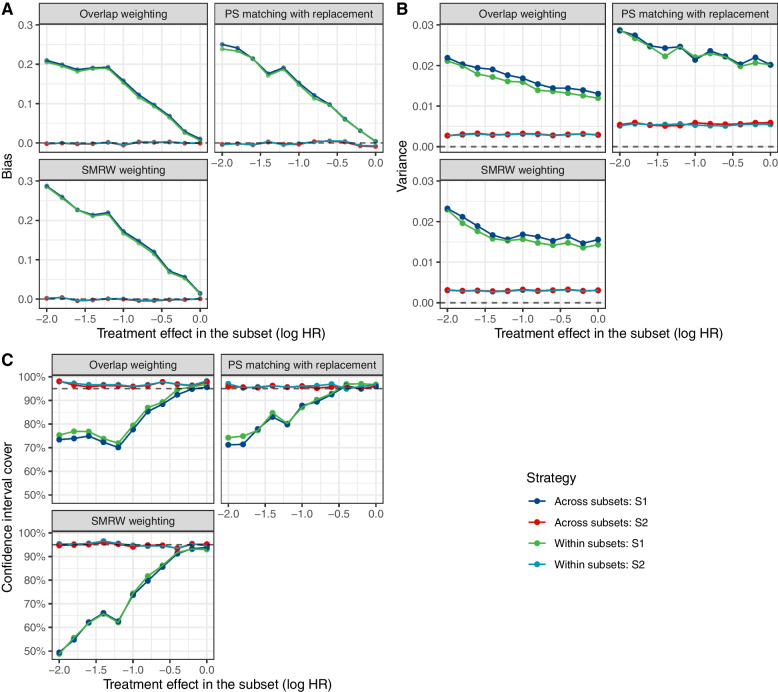
Bias according to the treatment effect in subset 1 (Scenario 2). Comparison of the “across subsets” or “within subsets” strategy in terms of the bias (**A**) variance (**B**) and the coverage of the 95% CI (**C**) for the estimation of the treatment effect in each subset

We first considered samples of $n=3,000$ individuals. In Scenario 1, where some treatment-by-subset interaction was introduced, both the “across subsets” and “within subsets” strategies yielded similar results, except using the “across subsets” approach when no interaction term was included in the PS model. Using this PS model, an important bias in the estimation of the treatment effect and impaired coverage in subset 1 were observed (Fig. 1). As expected, the inclusion of an instrumental variable in the PS model increased the variance in the estimation (Fig. 1 and Supplementary Fig. 3, Additional file 3). Bias in the estimated effect was higher in subset 1 than in subset 2 and was proportional to the treatment effect (Fig. 2).

**Fig. 3:**
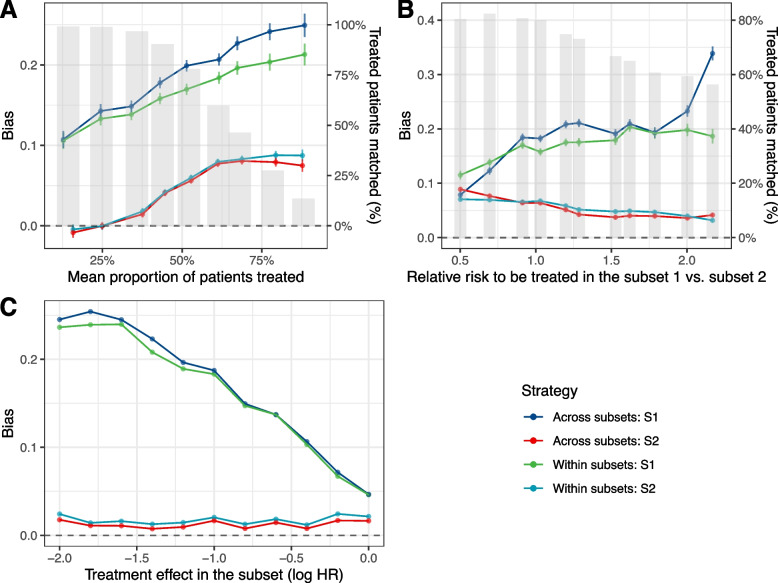
Bias in the estimation of the treatment effect under PS matching without replacement using the “across subsets” or the “within subsets” strategy, according to the treatment prevalence (**A**), the relative risk to be treated in subset 1 (**B**) and the treatment effect in subset 1 (**C**) (Scenario 1)

Using PS matching, caliper set at 0.1 gave similar results. We further show only results with the caliper set at 0.2 standard deviations of the logit of the PS. PS matching without replacement resulted in greater amounts of bias than the other approaches. This result can be explained by the discarding of treated patients because of the lack of comparative untreated patients. This bias was thus inversely proportional to the proportion of treated patients who could be matched in both strategies and inversely proportional to the relative number of comparative untreated patients (Fig. 3). When the relative risk to be treated increased in the small subset (subset 1), the “across subsets” strategy was significantly biased compared to the “within subsets” strategy using PS matching without replacement; however, this bias was controlled with replacement or PS-weighting methods (Supplementary Fig. 4B, Additional file 3). Given the importance of this bias, PS matching without replacement was not represented in the following simulations. The results of the simulations with this method can be found in Supplementary Fig. 5, Additional file 3.

When sample size decreased down to $n=300$, convergence issues occurred, notably using SMRW, while variance inflated using other methods, especially with the “within subets” strategy, which also reflects a convergence problem even if an estimation of the treatment effect could be obtained (Supplementary Figs. 6-8, Additional file 3). When the sample size increased from 300 to 5,000, results were poorly affected, except that PS matching without replacement achieved a decrease in variance while the bias persisted, resulting in a lowered coverage probability of confidence interval (Supplementary Fig. 9, Additional file 3), while type I error rate slightly decreased (Supplementary Fig. 5, Additional file 3). Otherwise, results were not markedly impacted by the size of the subsets (Supplementary Fig. 10, Additional file 3), the prognostic value of the subsets (Supplementary Fig. 11, Additional file 3), or by the treatment prevalence (Supplementary Fig. 4, Additional file 3).

**Fig. 4:**
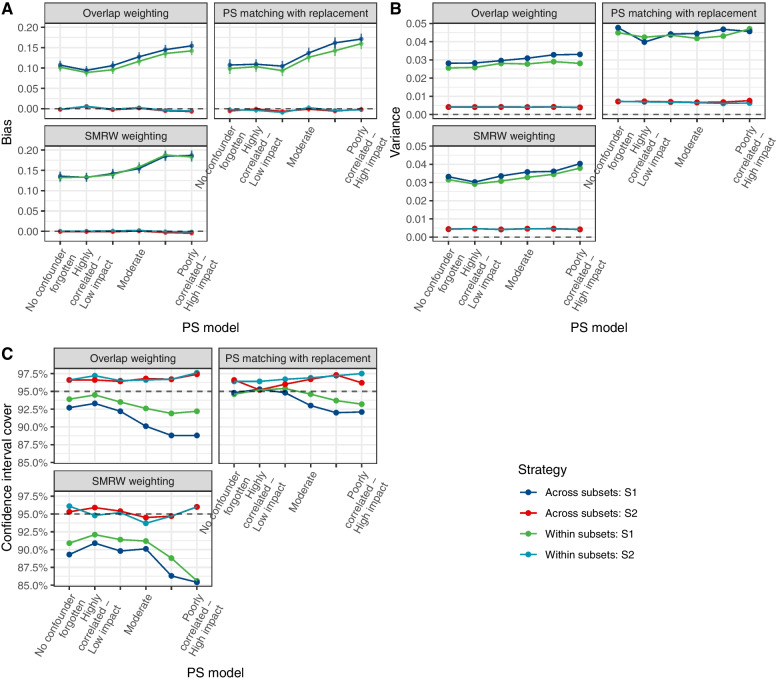
Simulations with an unknown confounder. Comparison of the “across subsets” or “within subsets” strategy in terms of bias (**A**), variance (**B**) and coverage (**C**) in the estimation of the treatment’s effect according to the presence of an unknown confounder (Scenario 1)

When a non-observed confounder was generated, all methods were biased, as expected. Bias was proportional to the impact of confounders on the outcome and inversely proportional to its correlation with an observed covariate (Fig. 4). This also resulted in a decrease of the coverage probability of the confidence interval, more pronounced with the “within subsets” approach. Overall, the overlap weighting and matching with replacement were slightly more robust than the SMRW weighting.

**Fig. 5:**
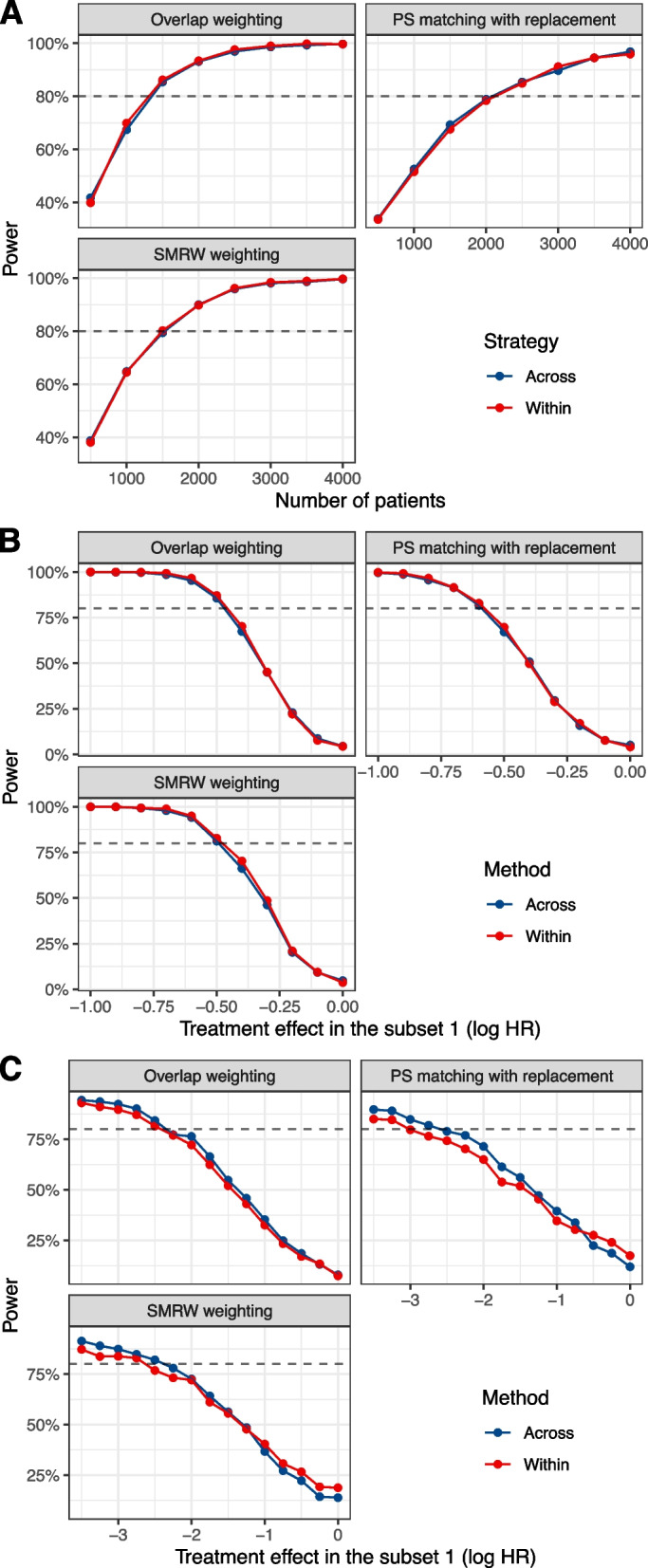
Power of the interaction test. Comparison of the power of the Gail and Simon quantitative interaction test by the number of patients (**A**) (Scenario 1) or the treatment effect (**B** and **C**) (Scenario 2). The number of patients is set to *n*=3000 in B and *n*=300 in C (Scenario 1). Robust estimate of variance was used for SMRW weighting when $n=300$, rather than bootstrapping, due to the importance of convergence problems that made it impratical to compute it

In the absence of treatment-by-subset interactions, whichever there was a treatment effect or not (Scenarios 3 and 4), type I error of the Gail & Simon interaction quantitative test was maintained (Supplementary Fig. 12, Additional file 3). However, the “across subsets” strategy appeared to be slightly more powerful for detecting an interaction in small samples (Fig. 5 C). Weighting methods (overlap weighting and SMRW weighting) also seemed to be more powerful than PS matching with replacement (Fig. 5).

## Revisited motivating example

### Methods

We applied similar methods as in the simulation study. The PS, defined as the probability of receiving a nerve resection, was estimated by a multivariable logistic regression model, including age at diagnosis, sex, tumor size (with log transformation), extraparenchymal invasion, skin or bone invasion, cN status, M stage, histological grade, histological type (adenoid cystic carcinoma or not), whether a total parotidectomy was performed, and whether a neck dissection was performed. These variables were chosen because of their known prognostic value and were measured before or at the time of the treatment choice. The T stage was not included in this main analysis because facial nerve invasion classifies the tumor as T4, thus almost consistently resulting in the resection of the facial nerve.

Regardless of the approach, in the matched or in the weighted pseudopopulations, the quality of the balance between the treatment groups was measured using the SMDs of potential confounders and of PS and based on the overlap coefficients (OVL) [26].

Interaction terms and/or quadratic terms were incorporated into the PS until a satisfactory balance was achieved. To display the SMDs in both subsets for each confounder, the connect-S plot proposed by Yang et al. [27] was used.

To address missing data, we performed multiple imputation with chained equations. We imputed 33 datasets, with 20 iterations, using an imputation model including important variables, the estimated cumulative baseline hazard based on the Nelson-Aalen estimator and interaction terms between the Nelson-Aalen estimator and covariates [28] (details are provided in Additional file 2). To account for multiple imputations, variances in estimated treatment effects were calculated by bootstrap [29], except for matching without replacement [30].

### Results

All PS models are described in Additional file 5. For the “across subsets” strategy, we additionally included in the PS model multiple interaction terms between the subset of interest (pretreatment facial palsy) and the prognostic covariates, whose effect on treatment choice was potentially modified by the existence of preoperative facial palsy (i.e., tumor grade, adenoid cystic carcinoma, extraparenchymal invasion, and bone or skin invasion).

**Fig. 6:**
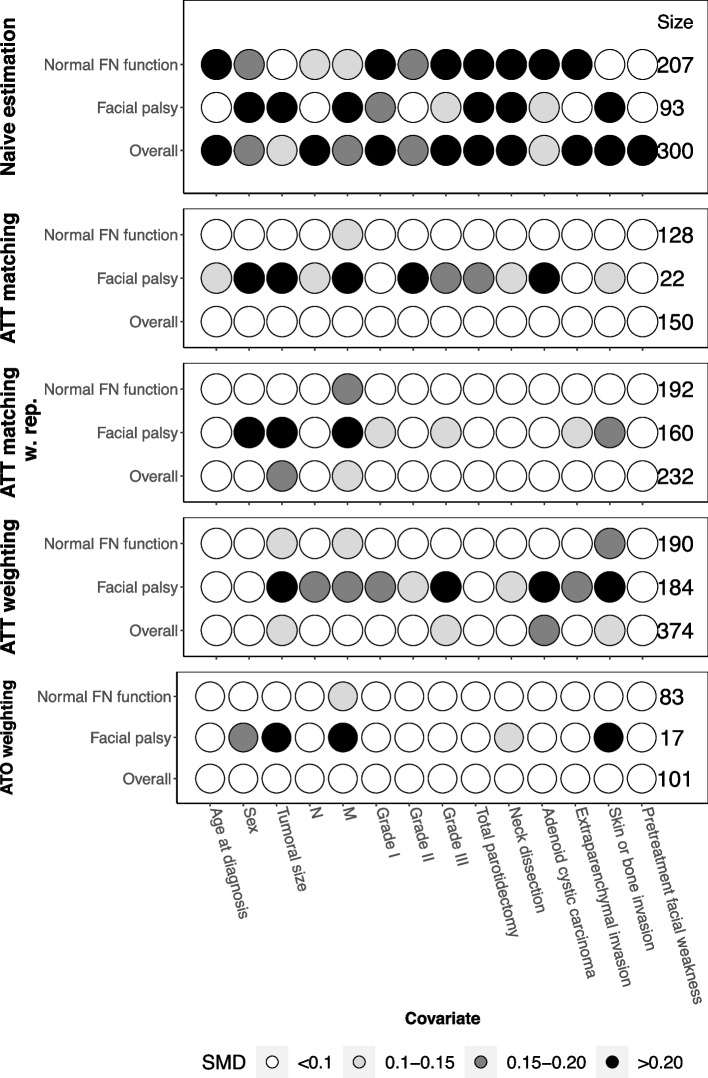
REFCOR data: Connect-S-plot with the “across subsets” approach. Connect-S plot representing standardized mean differences (SMDs) between treatment groups in the “across subsets” approach, in the original dataset ("naive estimation") and according to the PS-based method

**Fig. 7:**
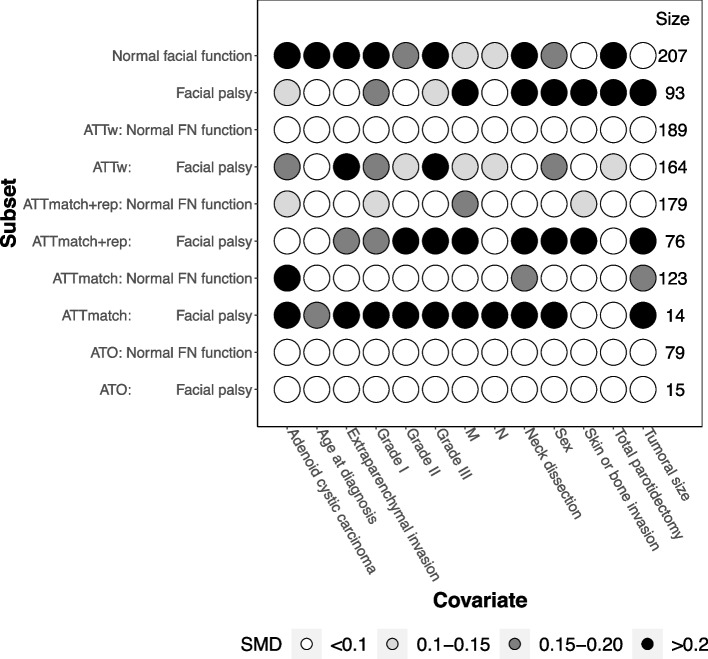
REFCOR data: Connect-S-plot with the “within subsets” approach. Connect-S-plot representing standardized mean differences (SMDs) between treatment groups in the “within subsets” approach in the original dataset (first two lines) and according to the PS-based method

**Fig. 8:**
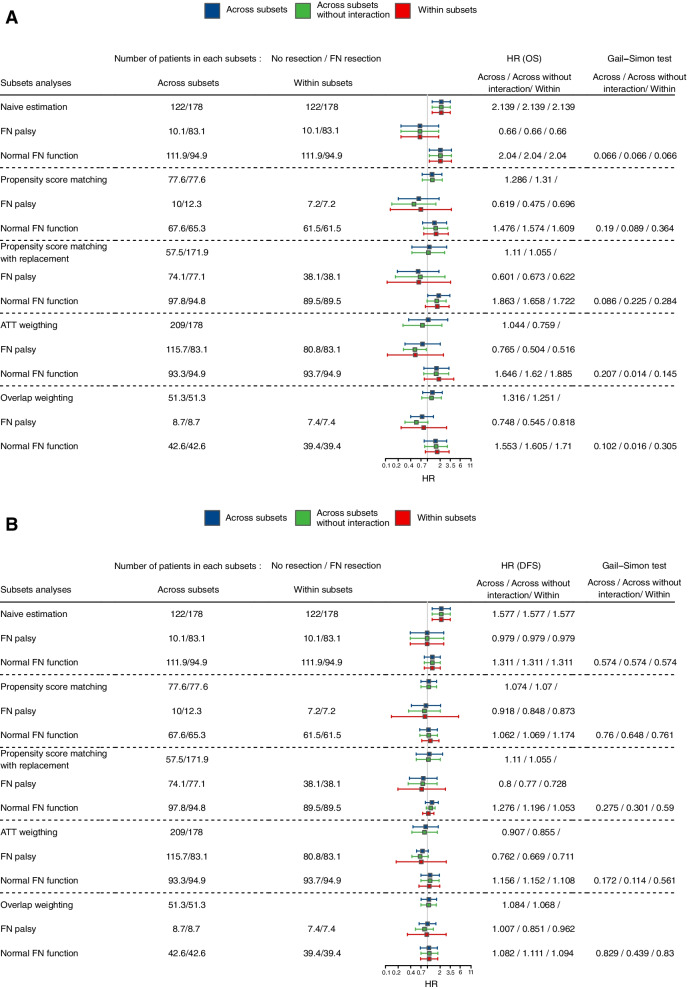
REFCOR data: Forest plots. Forest plots representing treatment effects in the subsets using the “across subsets” strategy with or without interaction terms and with the “within subsets” strategy. HR of death (**A**) and event (**B**) regarding facial nerve resection are represented. The first three lines refer to naive analyses performed on the original samples, ignoring potential confounding-by-indication bias. HR = hazard ratio. DFS = disease-free survival

Balances of covariates across treatment groups in each subset were more easily achieved with the “across subsets” strategy (Fig. 6 and Supplementary Figs. 11 to 14, Additional file 5) than with the “within subsets” strategy (Fig. 7 and Supplementary Fig. 15 to 22). Compared to the “within subsets” strategy, in the facial palsy subset, the “across subsets” strategy allowed us to include 18-22% more patients with PS weighting methods (means of 201.5 and 17.2 for weighted patients with SMRW and overlap weights, respectively, vs. 164.8 and 14.6 for the “within subsets” strategy), 62% more patients with the PS matching method (means of 22.4 vs. 13.8 patients) and 85% more patients with PS matching with replacement (means of 152.8 vs. 82.4 patients) (Fig. 8).

The treatment effects in both subsets obtained with the different PS-based methods are summarized in Fig. 8 for OS and DFS. No treatment-by-subset interaction was found regardless of the PS estimation strategy and the PS method with the recommended methods, but the interaction was significant when using the biased “across” method without an interaction term, using PS weighting methods. We used previously simulated data to obtain further insights into these results. To demonstrate a difference in our illustrative example, we used the Gail & Simon interaction quantitative test, which showed that 1,300 to 2,000 patients would have been required to demonstrate an interaction between subset 1 with log(HR) = -0.7 and subset 2 with log(HR) = 0 on the outcome, with a power of 80%. Otherwise, a log (HR) of -2 to -3 in subset 1 was also needed, depending on methods, to demonstrate an interaction with only 300 patients (Fig. 5).

## Discussion

In this study, we considered the issue of using propensity scores to estimate the heterogeneity in the treatment effect across baseline subsets. To address this issue, two strategies for estimating the propensity score were compared.

The first strategy consisted of estimating the propensity score on the whole sample, incorporating the subset variable, to create either a matched population or a pseudo population according to the PS-based method used. The treatment-by-subset interaction was then studied in the resulting whole matched or weighted sample. This strategy is theoretically valid because when the population is balanced on the true propensity score, the subsets are also theoretically balanced on treatment groups, as previously demonstrated [31]. However, in real life, the true propensity score is not known and must be estimated from the sample. This strategy can therefore lead to a poor balance between treatment groups in the covariates within subsets or even to a worsening of this imbalance [15]. In our illustrative case, this strategy afforded a good balance of covariates overall, although some imbalances persisted across treatment groups in the subsets (Figs. 6 and 7). Expectedly, most persisted differences were found in the small subset of patients with facial palsy (ranging from 93 original observations down to 17 in the overlap population with the “across subsets” strategy and 15 with the “within subsets” strategy).

The second strategy consisted of estimating the propensity score within the subsets, separately. The propensity scores were then used to create a matched population or a pseudo population in each subset, allowing the treatment effect to be evaluated in each subset separately. Then, treatment-by-subset interaction can be tested using the Gail and Simon statistics. This strategy, which should make it easier to obtain a balance in each subset, as previously demonstrated [15], did not work well in the case of our illustrative example. This result is likely because one subset had few patients, particularly in the case of PS matching without replacement, which suffered even more than the “across subsets” strategy from the limitations of adopting this approach for small samples [32].

Our simulation study showed that the two “across subsets” and “within subsets” strategies achieve similar results in terms of bias and variance, provided that interaction terms between the subset variable and other covariates influencing the choice of treatment are incorporated. Otherwise, the omission of these interaction terms based on the “across subsets” strategy induced an important bias, regardless of the PS-based method used, which confirms previous results [33, 34]. This bias led to the identification of an interaction that was not found with the other two strategies in our illustrative example. Interestingly, the incorporation of interaction terms that do not exist did not induce bias and only slightly increased the variance. Thus, when using the “across subsets” strategy, these results encourage the nonparsimonious use of interaction terms with the subset of interest. The demonstration of an interaction was also slightly more powerful when using the “across subsets” strategy in the case of a very small sample. These results were confirmed in our illustrative example, in which we found similar treatment effect estimates between methods but with lower variances using the “across subsets” strategy.

Focusing on the covariates included in the PS model, we confirmed that the use of an instrumental variable is detrimental in terms of variance. In contrast, the incorporation of a prognostic variable had little impact on the estimation of the treatment effect. However, the omission of a confounder led to a bias. Our study demonstrated that this bias was less important when matching with replacement or when overlap weight methods were used than when SMRW weighting was used. The “within subsets” strategy was also slightly more robust than the “across subsets” strategy in this case. Although previous studies on this topic focused on PS matching without replacement [14–16, 33–37], compared to the other methods, this method achieved a bias in the estimation of the treatment effect in our setting of large differences between subsets. This bias has been previously named the “unmatched patient bias” [38]. In the case of a small sample size, replacement has been demonstrated to reduce this bias [39]; we indeed found that this bias was proportional to the proportion of matched patients.

Our study has some limitations. First, we used propensity-score methods, while they could be outperformed by g-computation and/or doubly robust estimators [40, 41]. Nevertheless, we were only concerned by examining two main issues (imbalanced subgroups, right-censored outcomes) when implementing pre-specified subgroup analyses in a causal inference framework using propensity score approaches. Actually, we placed ourselves in the most popular setting in the medical and surgical literature for evaluating causal effects in observational studies, that is, targeting the ATT. We first used propensity score matching, in line with recent works that used Monte Carlo simulations to evaluate propensity score matching with data from complex sample surveys [42], when dealing with clustered data [43], or when a confounder has missing data [44]. Other PS-based methods could have been used, such as the inverse probability treatment weighting (IPTW), which is commonly used in subset analyses [16, 33, 36, 37]. This method has been reported to achieve better performance than PS matching in the case of right-censored outcomes [16]. In secondary analyses, we thus also used IPTW using either standardized mortality ratio weights or overlap weights [24]. Of note, the later targets another estimand, the ATO. Actually, the ATO targets an “artificial” and less defined population consisting of patients with the highest mutual overlap of PS between the 2 treatment groups. ATO can be considered as an intermediate between the average treatment effect (ATE) and the ATT. The population targeted by the ATO indeed consists of patients with a high probability of appearing in either of the 2 treatment groups, that could be interpreted as a population at clinical equipoise. This complicated interpretation is the main drawback of such overlap weighting. Nevertheless, the overlap weights facilitate a perfect and straight balance between groups and could therefore be largely used in this setting where it is difficult to obtain a satisfactory balance with other methods. Otherwise, overlap weighting has been shown to preserve a higher proportion of the sample with a reduction in bias [24] and to provide close performances to that of g-computation [8]. However, the overlap weighting method did not outperform other PS-based methods in our simulation study.

Second, we considered only interactions between the subset and covariates that affected treatment choice rather than the outcome. However, the omission of an interaction term when there is an interaction between the subset and prognostic covariate has already been reported to bias the treatment effect [33].

Third, we did not study other alternatives to the multivariable logistic model that have been proposed. These alternatives include the use of a generalized propensity score [45] or a balancing propensity score [31], extending the covariate balancing propensity score [46] for multiple subset analyses; however, given the small difference observed between the two abovementioned strategies, we did not evaluate them.

## Conclusions

In conclusion, when aiming to evaluate the treatment effect in prespecified subsets from observational data using propensity score approaches, estimating the propensity score in the whole sample appears a valid option compared to the estimation of the propensity score within each subset, provided that interaction terms between the subsets and other covariates are included in the PS model. This “across subsets” strategy could be useful in small samples, especially when the samples are imbalanced in terms of the subsets. Indeed, in this setting, estimating the propensity score can lead to convergence issues in a small subset while preventing a satisfactory balance between treatment groups. Weighting methods appear to be more powerful for demonstrating a treatment-by-subset interaction. In the case of PS matching, the use of replacement appears to be preferred in this setup with a lack of comparable patients, regardless of the PS estimation strategy.

### Supplementary information

**Additional file 1.**

**Additional file 2.**

**Additional file 3.**

**Additional file 4.**

**Additional file 5.**

**Additional file 6.**

## Data Availability

The data that illustrate this study are available from the REFCOR but restrictions apply to the availability of these data, which were used under license for the current study, and so are not publicly available. Data are however available from the authors upon reasonable request and with permission of the REFCOR by contacting the scientific committee (benjamin.verillaud@aphp.fr).

## Abbreviations

ATE: Average treatment effect
ATO: Average treatment effect in the overlap
ATT: Average treatment effect in the treated
cN+: Clinically involved lymph nodes
DFS: Disease-free survival
FN: Facial nerve
HR: Hazard-ratio
IPTW: Inverse probability treatment weighting
M1: Metastasis
OS: Overall survival
OVL: Overlap coefficient
PS: Propensity score
REFCOR: *Réseau d’expertise français sur les cancers ORL rares*
SD: Standard deviation
S1: Subset 1
S2: Subset 2
SMD: Standardized mean difference
SMRW: Standardized mortality ratio weight
