# Supplementary material for: How to perform prespecified subgroup analyses when using propensity score methods in the case of imbalanced subgroups
Source: BMC Med Res Methodol. 2023 Oct 31;23:255. doi: 10.1186/s12874-023-02071-8 (PMC10617117; doi:10.1186/s12874-023-02071-8)
Supplement: Supplementary file 1 — Additional file 1. [file 12874_2023_2071_MOESM1_ESM.pdf]

Additional file 1

Flow-chart

Data were obtained for 21 centers in the network, corresponding to 707 patients. Among these patients, 300 had a tumor in contact with the nerve, including 178 who benefited from a facial nerve resection (Figure 1). Of the 170 patients with a tumor in contact without resection, 92 had inframillimetric margins, 63 had macroscopic invasion, and 52 had perineural involvement. By taking one of the 3 criteria, 122 patients could be selected in the comparison group.

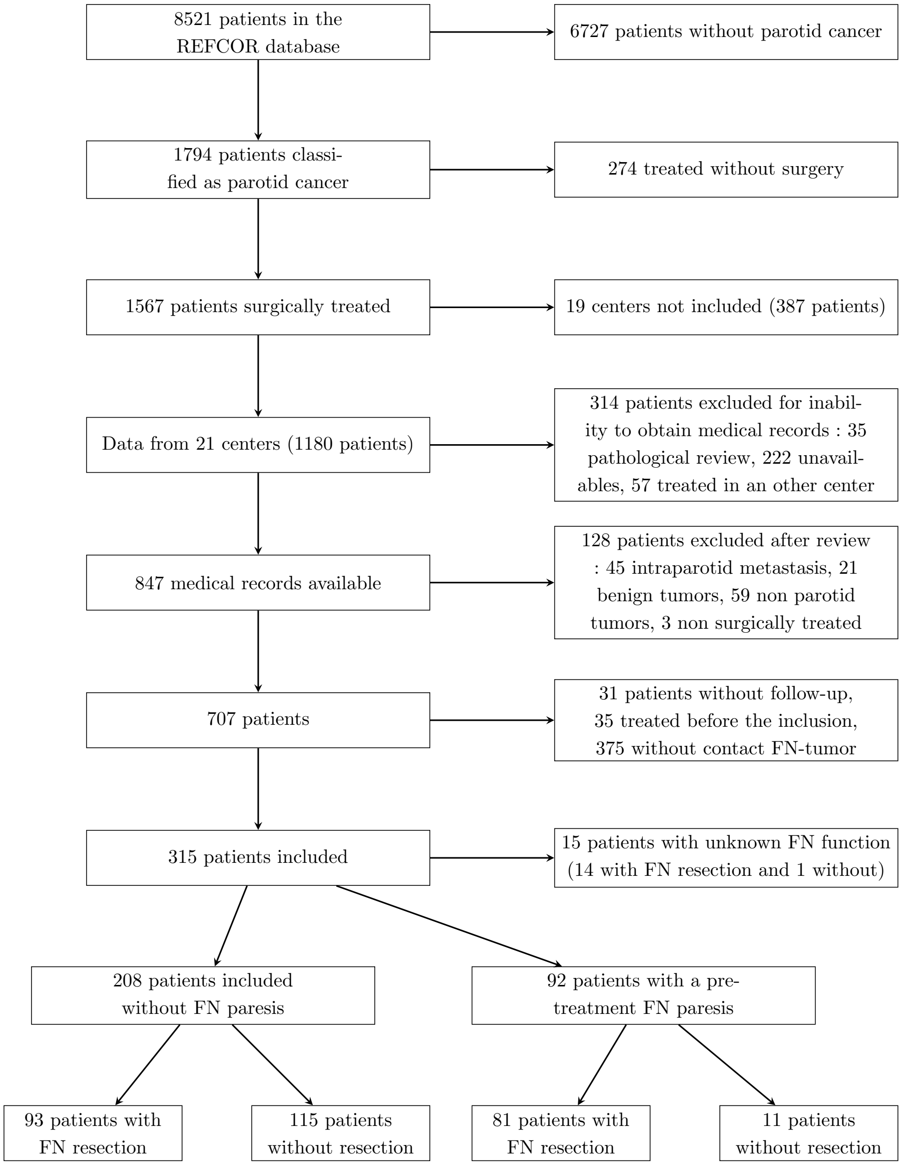

Supplementary Figure 1 Flow-chart of the study FN = facial nerve.
