# Supplementary material for: How to perform prespecified subgroup analyses when using propensity score methods in the case of imbalanced subgroups
Source: BMC Med Res Methodol. 2023 Oct 31;23:255. doi: 10.1186/s12874-023-02071-8 (PMC10617117; doi:10.1186/s12874-023-02071-8)
Supplement: Supplementary file 2 — Additional file 2. [file 12874_2023_2071_MOESM2_ESM.pdf]

Additional file 2

Missing data

Regarding the 300 patients included, with or without FN palsy, the missing data are summarized in figure 2.

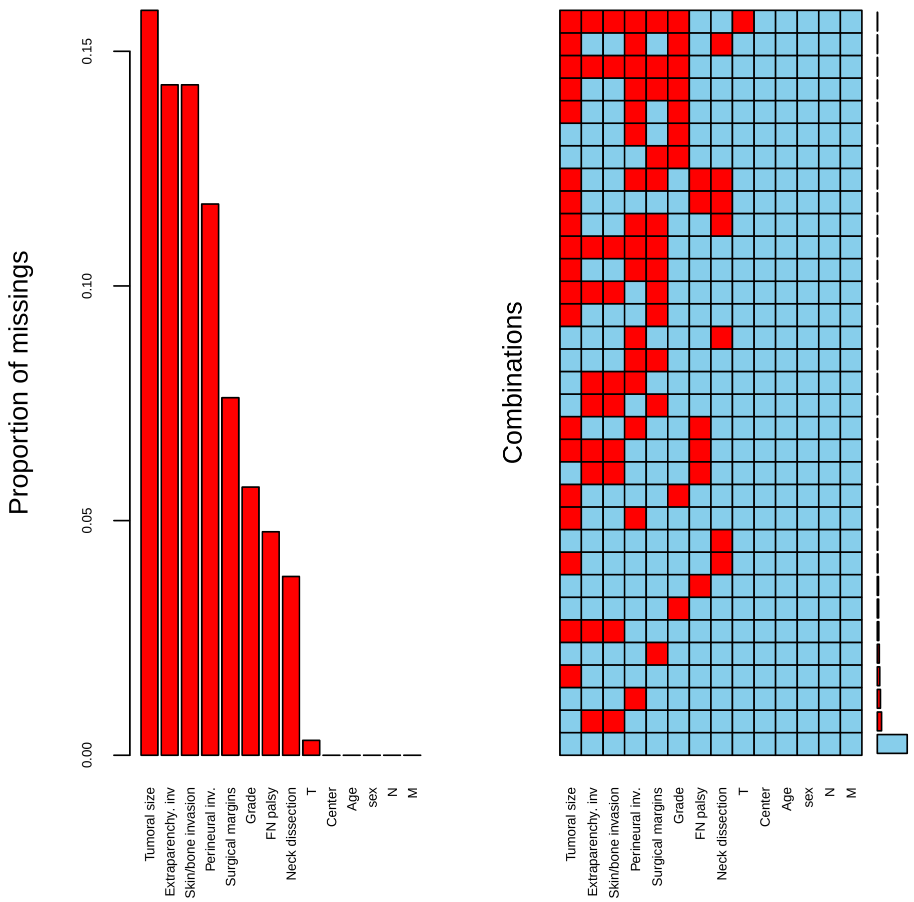

Supplementary Figure 2 Missing data in the 300 patients included. The proportion of missing data (left panel) and its pattern (right panel) are represented.

The complete case sample corresponded to 203 patients out of the 300 patients initially selected. Of these 203 patients, 110 underwent nerve resection, and 93 did not. The characteristics of the excluded patients are shown in table 1.

Methods

Given the existence of missing data and the non-MCAR mechanism (cf. Figure 2 and Table 1), we performed statistical analyses on the imputed datasets obtained after multiple imputation with chained equations.

A total of 33 imputed datasets were created using multiple imputations by chained equation algorithms with 20 iterations [1]. All variables considered in the propensity score model, important covariates linked to survival but not to treatment (surgical margins, histologically nodal status, use of radiotherapy or chemotherapy), and

Supplementary Table 1 Characteristics of the population according to the realization of a nerve resection.

	Complete cases	Missing data	SMD
n	203	97	
Age at diagnosis (mean (SD))	60.65 (16.55)	62.72 (16.11)	0.127
Male sex (%)	118 (58.1)	63 (64.9)	0.141
T (%)			0.791
T1	38 (18.7)	5 (5.2)	
T2	72 (35.5)	18 (18.6)	
T3	44 (21.7)	18 (18.6)	
T4	49 (24.1)	56 (57.7)	
N (%)			0.176
0	148 (72.9)	63 (64.9)	
1	10 (4.9)	6 (6.2)	
2	41 (20.2)	26 (26.8)	
3	4 (2.0)	2 (2.1)	
M1 (%)	13 (6.4)	7 (7.2)	0.032
Grade (%)			0.195
I	53 (26.1)	15 (18.5)	
II	27 (13.3)	10 (12.3)	
III	123 (60.6)	56 (69.1)	
Total parotidectomy (%)	188 (92.6)	79 (83.2)	0.293
Deep lobe tumor (%)	53 (26.1)	29 (33.7)	0.167
Tumoral size (mm)	29.38 (14.49)	36.04 (22.90)	0.347
Perineural invasion (%)	0.67 (0.47)	0.85 (0.36)	0.435
Neck dissection (%)	175 (86.2)	74 (86.0)	0.005
VanderPoorten Score	5.53 (1.12)	6.54 (1.17)	0.890
Prognostic index (%)			0.755
1	15 (8.7)	0 (0.0)	
2	37 (21.4)	2 (11.8)	
3	44 (25.4)	2 (11.8)	
4	77 (44.5)	13 (76.5)	

covariables linked to missing data (center of inclusion and date of inclusion), were plugged in the imputation model as well as the event indicator and the cumulative hazard, estimated with the Nelson-Aalen estimator. Interaction terms between the cumulative hazard and other covariates were also implemented [1]. Propensity scores were then estimated within each subset, and a treatment estimate was obtained for each imputed dataset [2]. Final estimations were obtained using Rubin's rules [3]. The number of patients in each subset was pooled following the same rules. The variance in the estimation was obtained by bootstrapping 1000 replicated datasets, followed by multiple imputation on each bootstrapped dataset with the same parameters as described above, before performing PS-based methods to obtain a treatment estimation [4]. Because bootstrapping is not adequate to estimate the variance in the case of matching with replacement, we used the variance estimator proposed by Austin and Caufri [5], pooled following Rubin's rules. Concerning the facial palsy subset, because a significant proportion of bootstrapped samples results in a convergence problem (only 376/1000 bootstrapped samples resulted in an estimation for PS matching in this subset), we used a robust variance pooled following Rubin's rules.

Author details

References

1. White IR, Royston P. Imputing missing covariate values for the Cox model. Statistics in Medicine. 2009;28(15):1982–1998.
2. Leyrat C, Seaman SR, White IR, Douglas I, Smeeth L, Kim J, et al. Propensity score analysis with partially observed covariates: How should multiple imputation be used? Statistical Methods in Medical Research. 2019;28(1):3–19.
3. White IR, Royston P, Wood AM. Multiple imputation using chained equations: Issues and guidance for practice. Statistics in Medicine. 2011;30(4):377–399.

4. Ling A, Montez-Rath M, Mathur M, Kapphahn K, Desai M. How to Apply Multiple Imputation in Propensity Score Matching with Partially Observed Confounders: A Simulation Study and Practical Recommendations. Journal of Modern Applied Statistical Methods. 2021;19(1):1.
5. Austin PC, Cafri G. Variance estimation when using propensity-score matching with replacement with survival or time-to-event outcomes. Statistics in Medicine. 2020;39(11):1623–1640.
