# Supplementary material for: How to perform prespecified subgroup analyses when using propensity score methods in the case of imbalanced subgroups
Source: BMC Med Res Methodol. 2023 Oct 31;23:255. doi: 10.1186/s12874-023-02071-8 (PMC10617117; doi:10.1186/s12874-023-02071-8)
Supplement: Supplementary file 3 — Additional file 3. [file 12874_2023_2071_MOESM3_ESM.pdf]

Additional file 3

Supplementary simulations

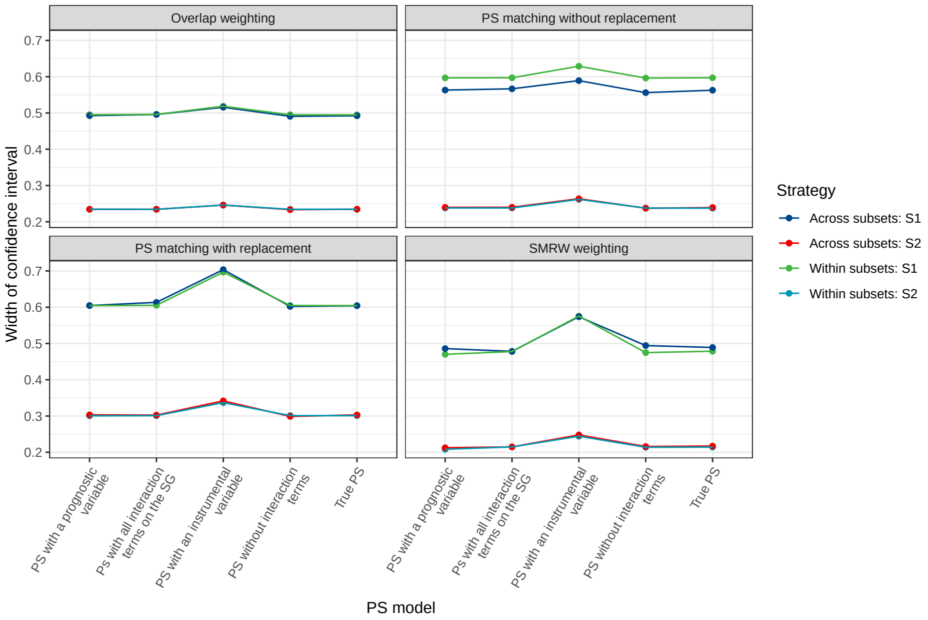

Supplementary Figure 3 Width of the confidence interval in the estimation of the treatment's effect according to the PS model used.

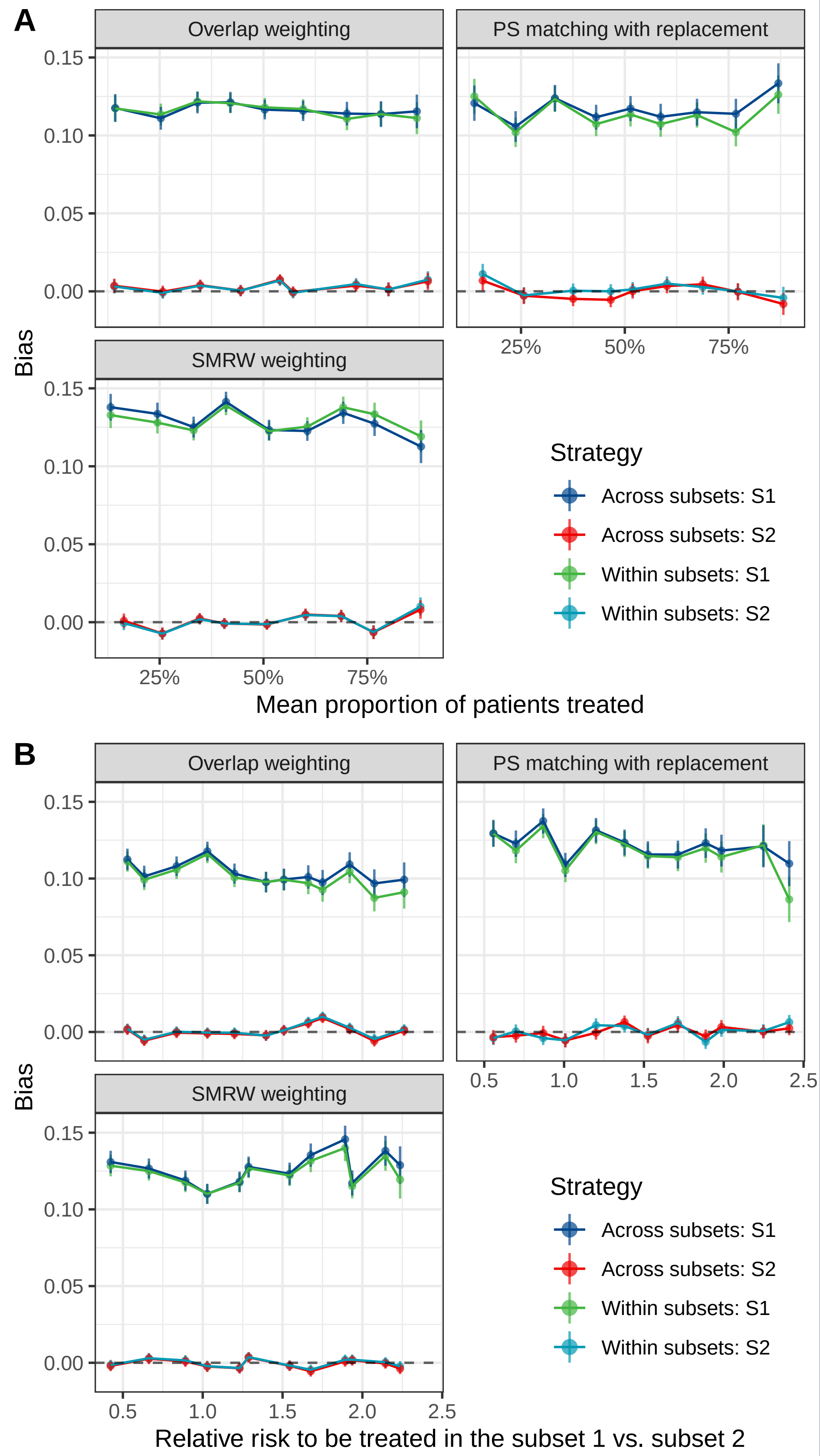

Supplementary Figure 4 Bias in the estimation of the treatment effect in each subset according to the relative number of treated patients in subset 1. Simulations according to the proportion of treated patients, when similar in both subsets (A) or according to the relative risk to be treated in subset 1 vs. subset 2 (B) (Scenario 1).

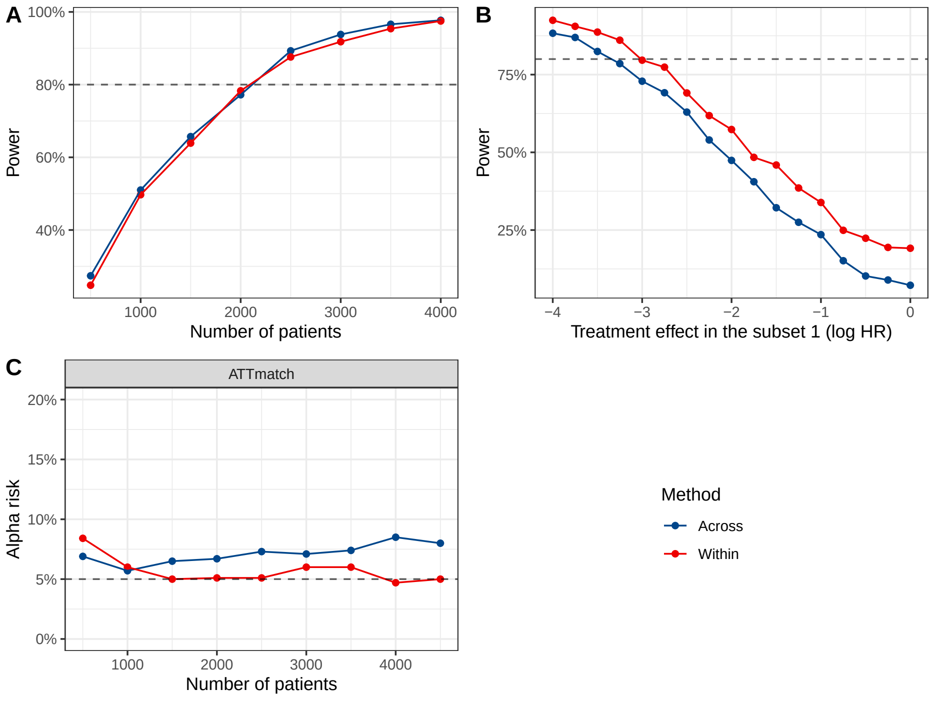

Supplementary Figure 5 Power (A and B) and alpha risk (C) of the Gail & Simon quantitative interaction test when using PS matching without replacement. Simulations according to the number of patients (A and C) or to the treatment effect in subset 1 (B). (Scenario 1 (A and B) and 4 (C)

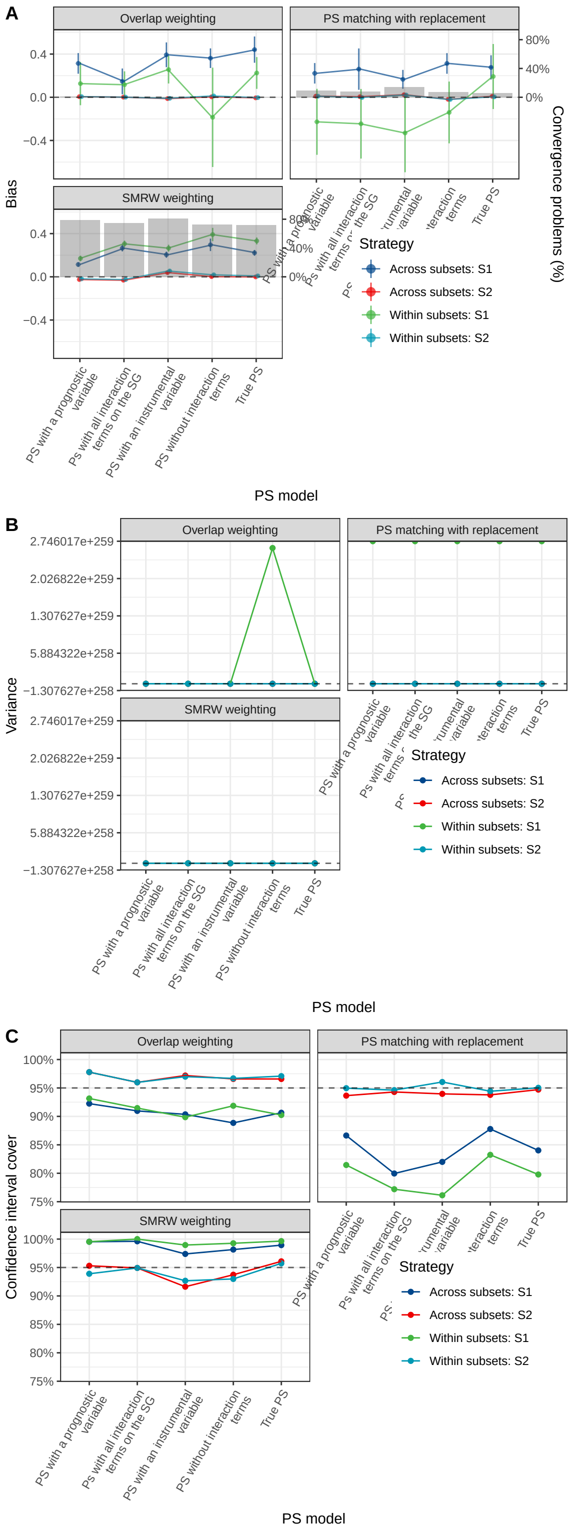

Supplementary Figure 6 Comparison of strategies according to the PS model with n=300
Comparison of "across subsets" or "within subsets" strategy in terms of the mean absolute bias (A), variance (B) and the coverage of the 95% CI (C) according to the PS model (Scenario 1). Frequency of convergences issues are represented by histograms (A). S1 = subset 1; S2 = subset 2.

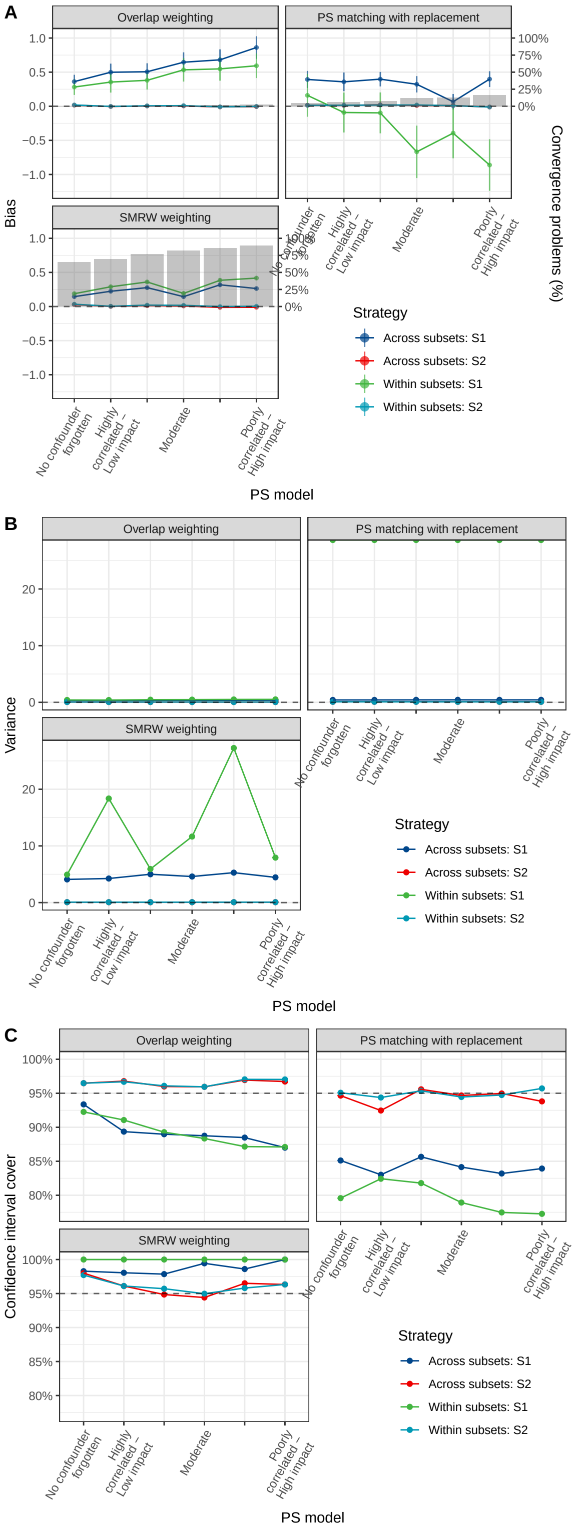

Supplementary Figure 7 Simulations with an unknown confounder when n=300 Comparison of the “across subsets” or “within subsets” strategy in terms of bias (A) variance (B) and coverage (C) in the estimation of the treatment’s effect according to the presence of an unknown confounder (Scenario 1). Frequency of convergences issues are represented by histograms (A).

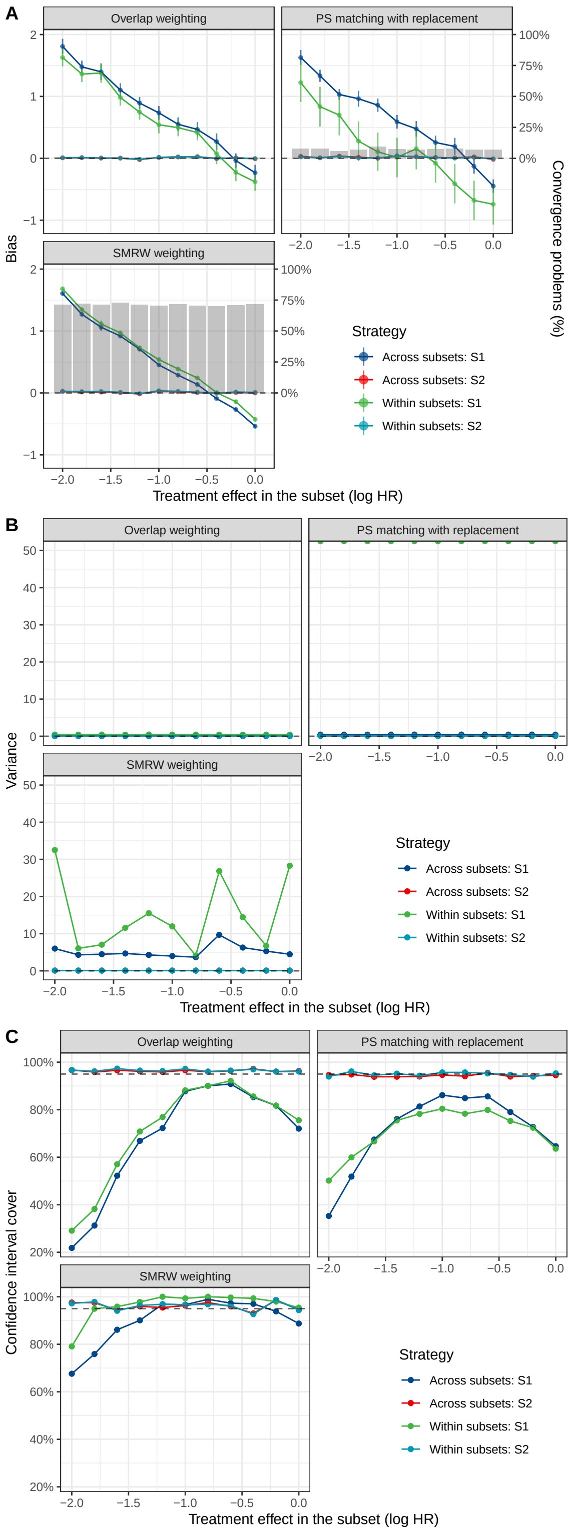

Supplementary Figure 8 Bias according to the treatment effect in subset 1, with n=300 (Scenario 2) Comparison of the "across subsets" or "within subsets" strategy in terms of the bias (A) variance (B) and the coverage of the 95% CI (C) for the estimation of the treatment effect in each subset. Frequency of convergences issues are represented by histograms (A).

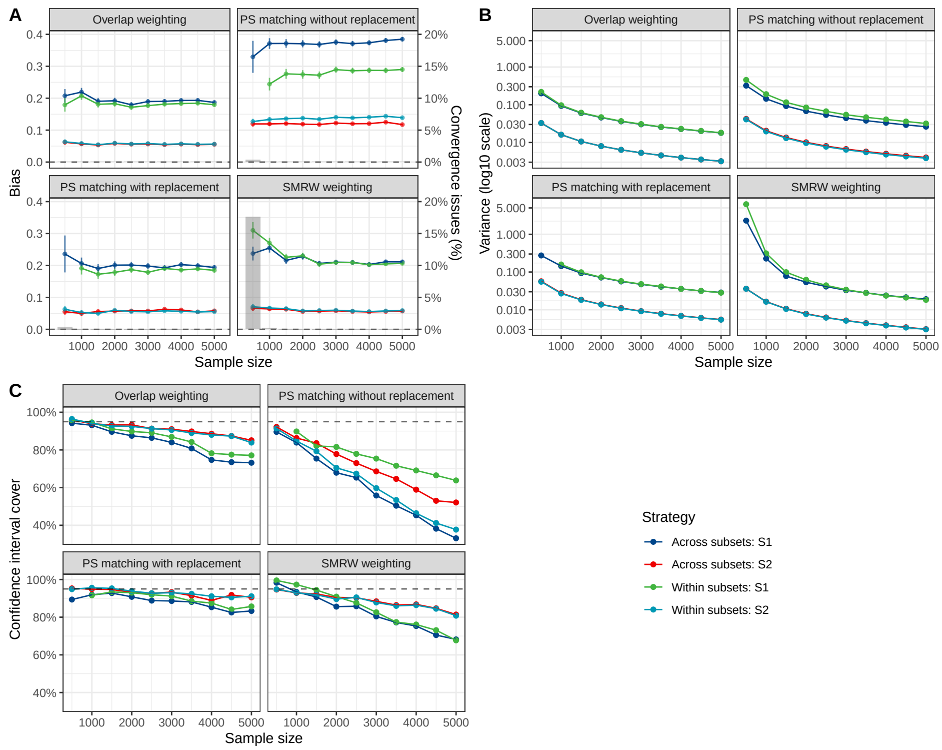

Supplementary Figure 9 Bias (A), variance (B) and coverage (C) in the estimation of the treatment's effect according to the number of patients in the overall population (Scenario 1). Frequency of convergences issues are represented by histograms (A)

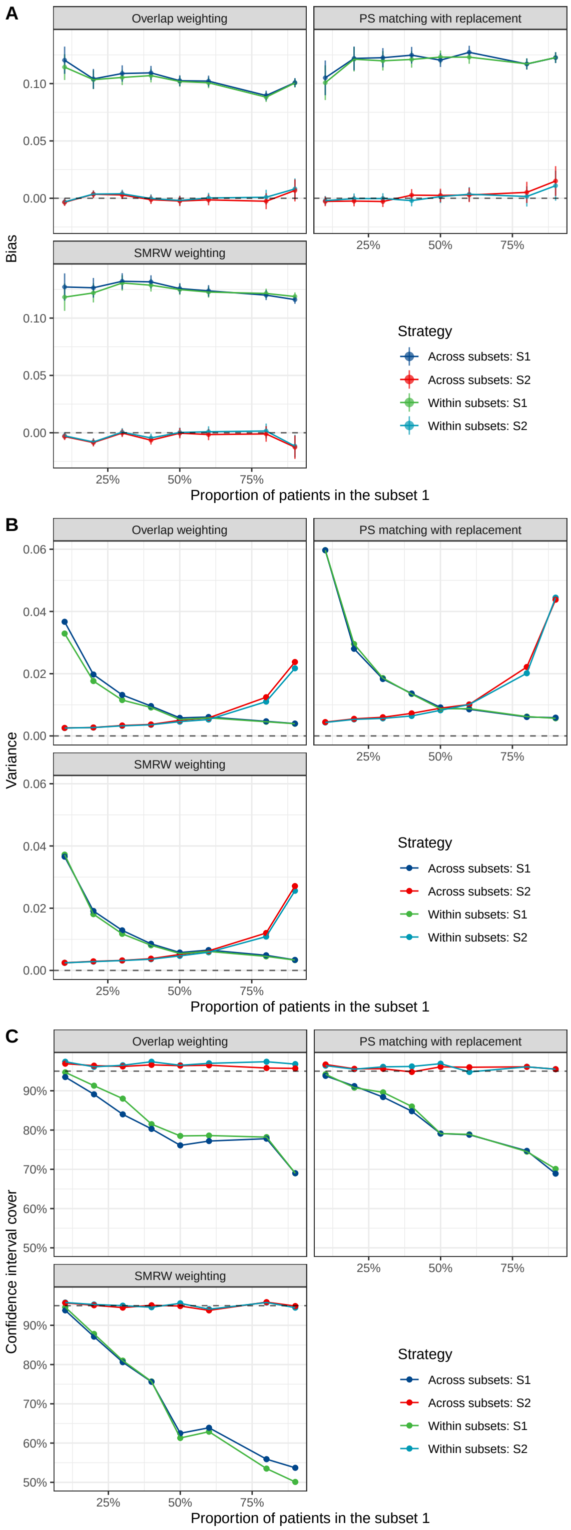

Supplementary Figure 10 Bias (A), variance (B) and coverage (C) in the estimation of the treatment effect according to the proportion of patients in subset 1 (Scenario 1)

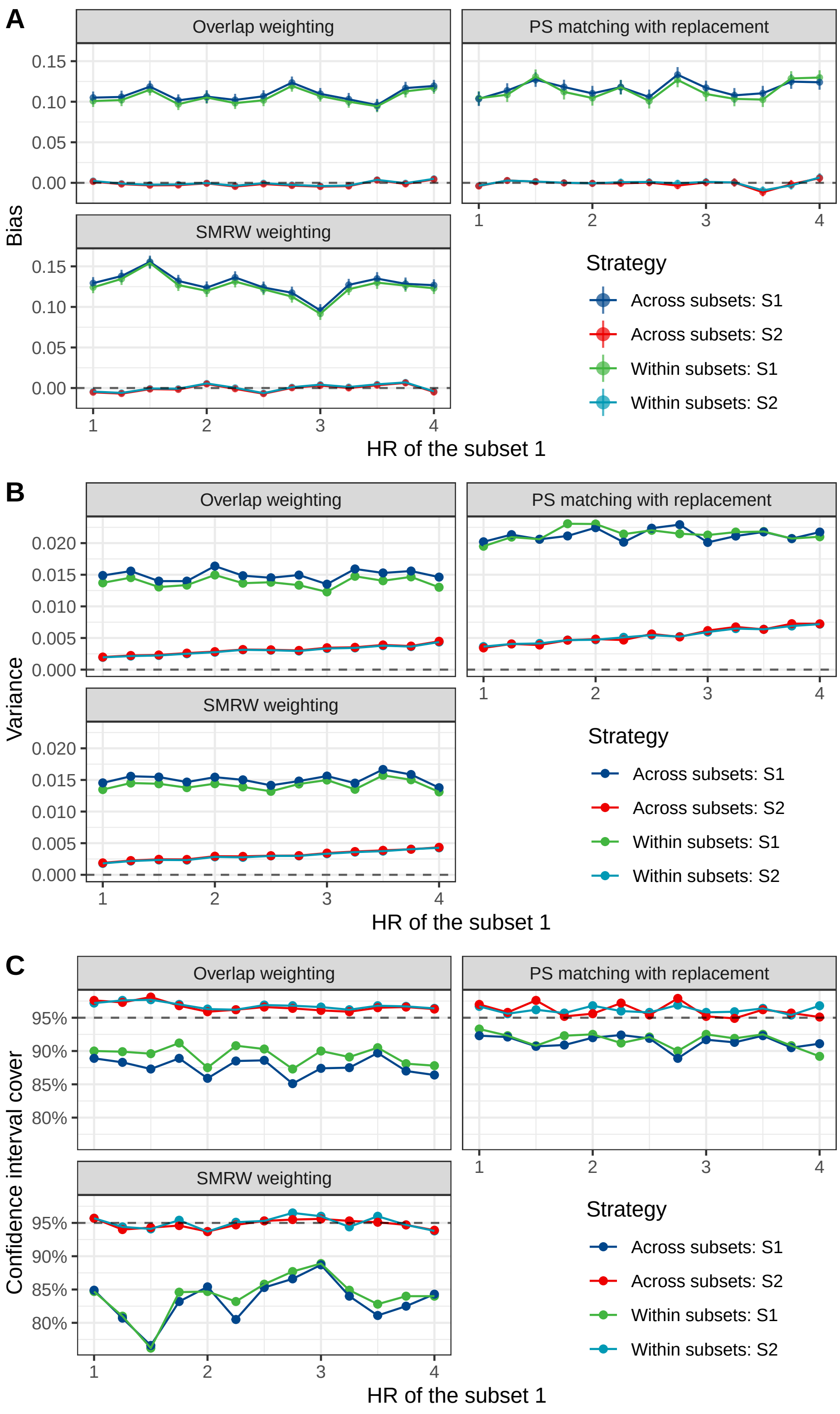

Supplementary Figure 11 **Bias (A) variance (B) and coverage (C) in the estimation of the treatment's effect according to the prognostic value of subset 1 compared to subset 2 (Scenario 1).**

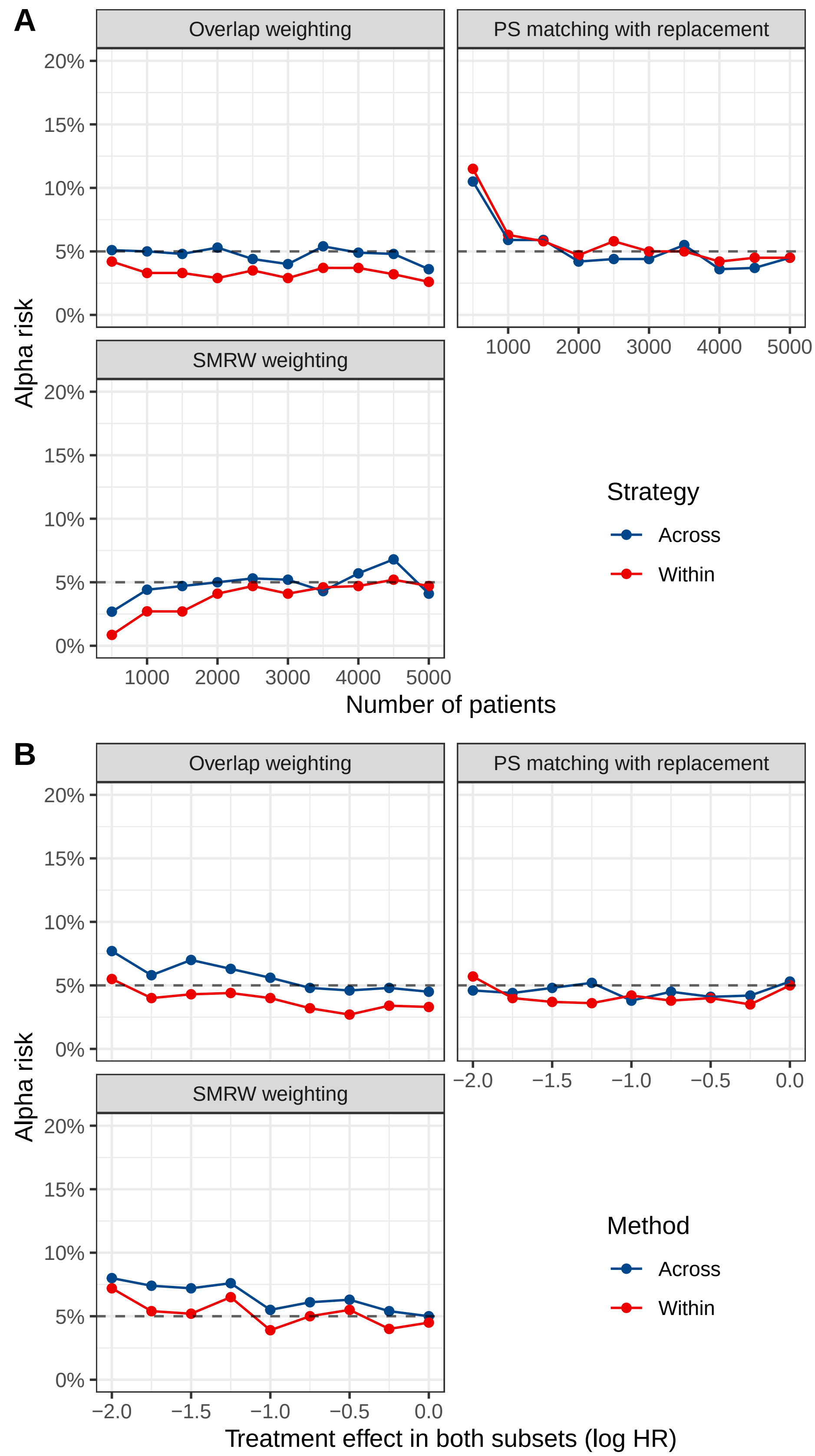

Supplementary Figure 12 Alpha risk of the Gail and Simon quantitative interaction test according to the number of patients (A) (Scenario 4) or to the treatment effect (B) (Scenario 3).
