# Supplementary material for: How to perform prespecified subgroup analyses when using propensity score methods in the case of imbalanced subgroups
Source: BMC Med Res Methodol. 2023 Oct 31;23:255. doi: 10.1186/s12874-023-02071-8 (PMC10617117; doi:10.1186/s12874-023-02071-8)
Supplement: Supplementary file 4 — Additional file 4. [file 12874_2023_2071_MOESM4_ESM.pdf]

Additional file 4

Naive analysis

Based on the original sample, deleterious outcomes were observed in the resection group, in terms of both overall survival (OS) and disease-free survival (DFS), with an estimated hazard ratio (HR) of 2.139, CI 95% = [1.295-3.531], p = 0.003 for OS and HR = 1.577, CI 95% = [1.102-2.258], p = 0.013 for DFS (figure 13). The HR for OS was estimated at 0.66 (CI 95% = [0.234-1.859]) for treated patients with FN palsy and 2.04 (CI 95%=[1.105-3.764]) for treated patients without FN palsy (p = 0.066 on the Gail & Simon quantitative interaction test). Concerning DFS, the HR was estimated at 0.979 (CI 95% = [0.391-2.455]) for treated patients with FN palsy and 1.311 (CI 95%=[0.847-2.027]) for treated patients without FN palsy (p = 0.574 on the Gail & Simon quantitative interaction test).

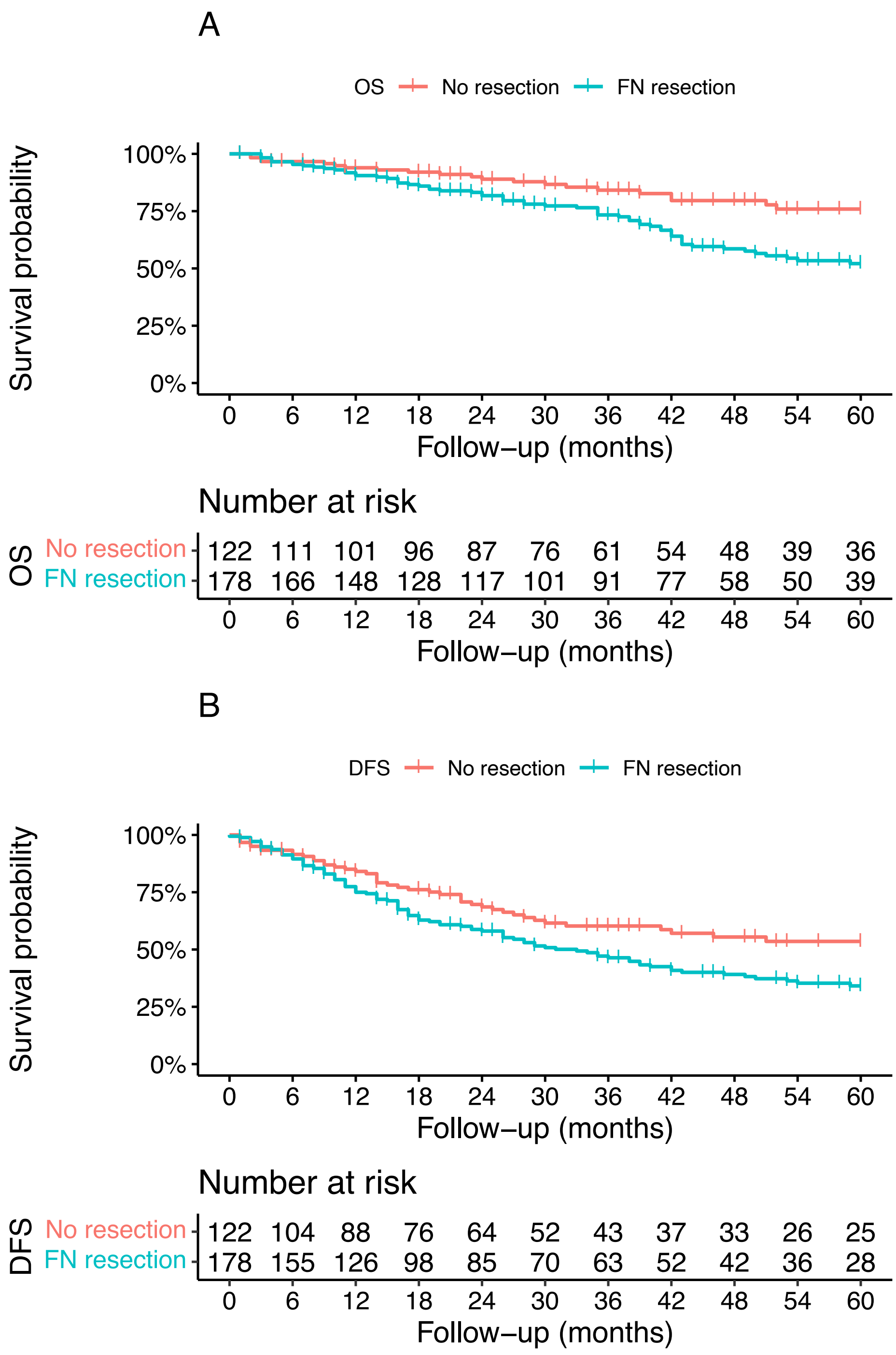

Supplementary Figure 13 **Overall survival (OS) (A) and disease-free survival (DFS) (B) according to the nerve resection group.**
