# Supplementary material for: How to perform prespecified subgroup analyses when using propensity score methods in the case of imbalanced subgroups
Source: BMC Med Res Methodol. 2023 Oct 31;23:255. doi: 10.1186/s12874-023-02071-8 (PMC10617117; doi:10.1186/s12874-023-02071-8)
Supplement: Supplementary file 5 — Additional file 5. [file 12874_2023_2071_MOESM5_ESM.pdf]

Additional file 5

"Across subsets" strategy

PS matching

The PS model used is $nerve\_resection* \sim age + age^2 + sex + tumoral\_size + log(tumoral\_size) + N + M + grade + total\_parotidectomy + neck\_dissection * + adenoid\_cystic\_carcinoma + extra\_parenchymal\_invasion + skin\_bone\_invasion + pretreatment\_facial\_palsy + pretreatment\_facial\_palsy : grade + grade : log(tumoral\_size) + pretreatment\_facial\_palsy : adenoid\_cystic\_carcinoma + pretreatment\_facial\_palsy : extra\_parenchymal\_invasion$

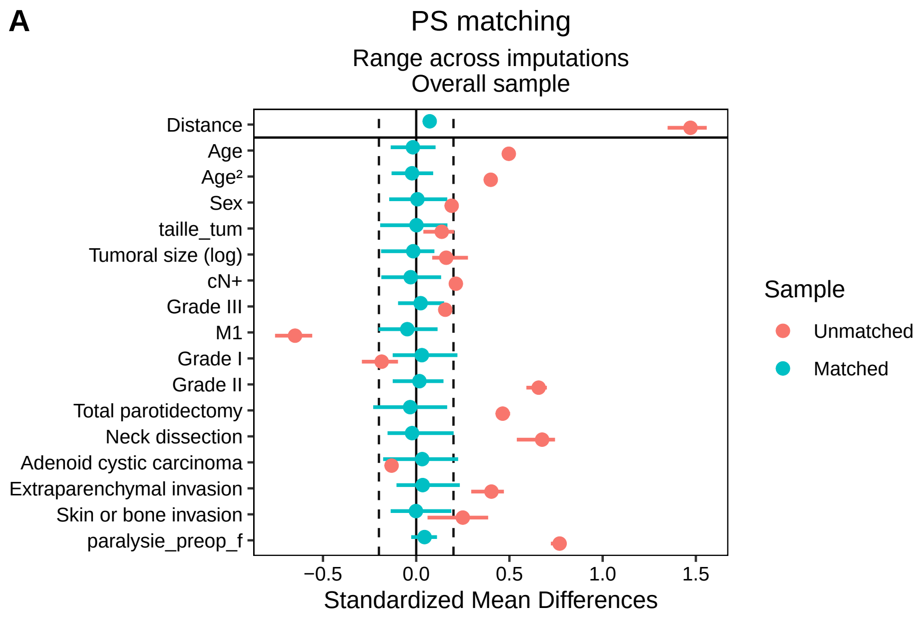

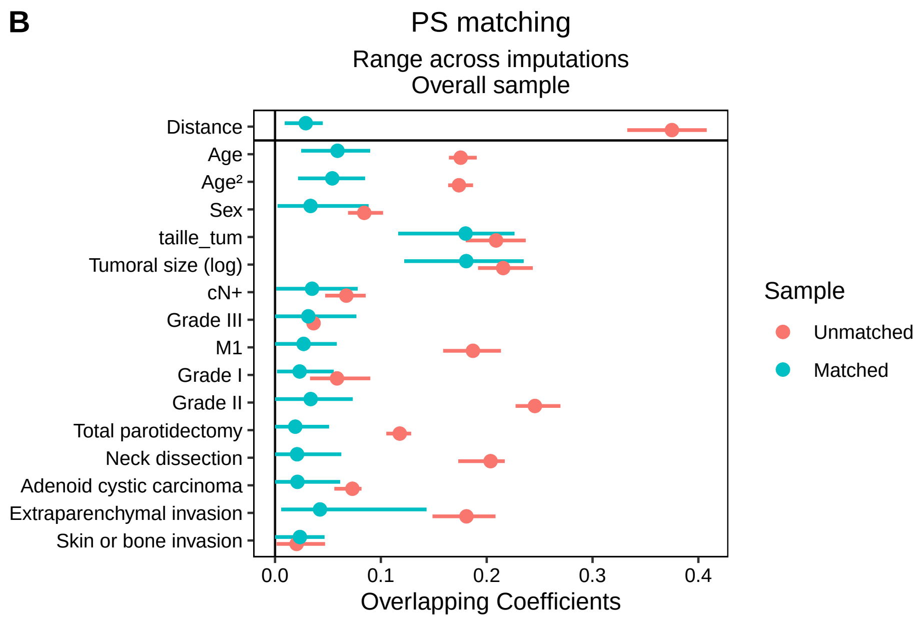

Supplementary Figure 14 Love plot of the balance of variables achieved before and after propensity score matching. Standardized mean differences (A) and overlapping coefficients (B) for each covariate are presented.

PS matching with replacement

The PS model used is $nerve\_resection* \sim age + age^2 + sex + tumoral\_size + log(tumoral\_size) + N + M + grade + total\_parotidectomy + neck\_dissection * +adenoid\_cystic\_carcinoma + extra\_parenchymal\_invasion + skin\_bone\_invasion + pretreatment\_facial\_palsy + pretreatment\_facial\_palsy : grade + grade : log(tumoral\_size) + pretreatment\_facial\_palsy : adenoid\_cystic\_carcinoma + pretreatment\_facial\_palsy : extra\_parenchymal\_invasion$

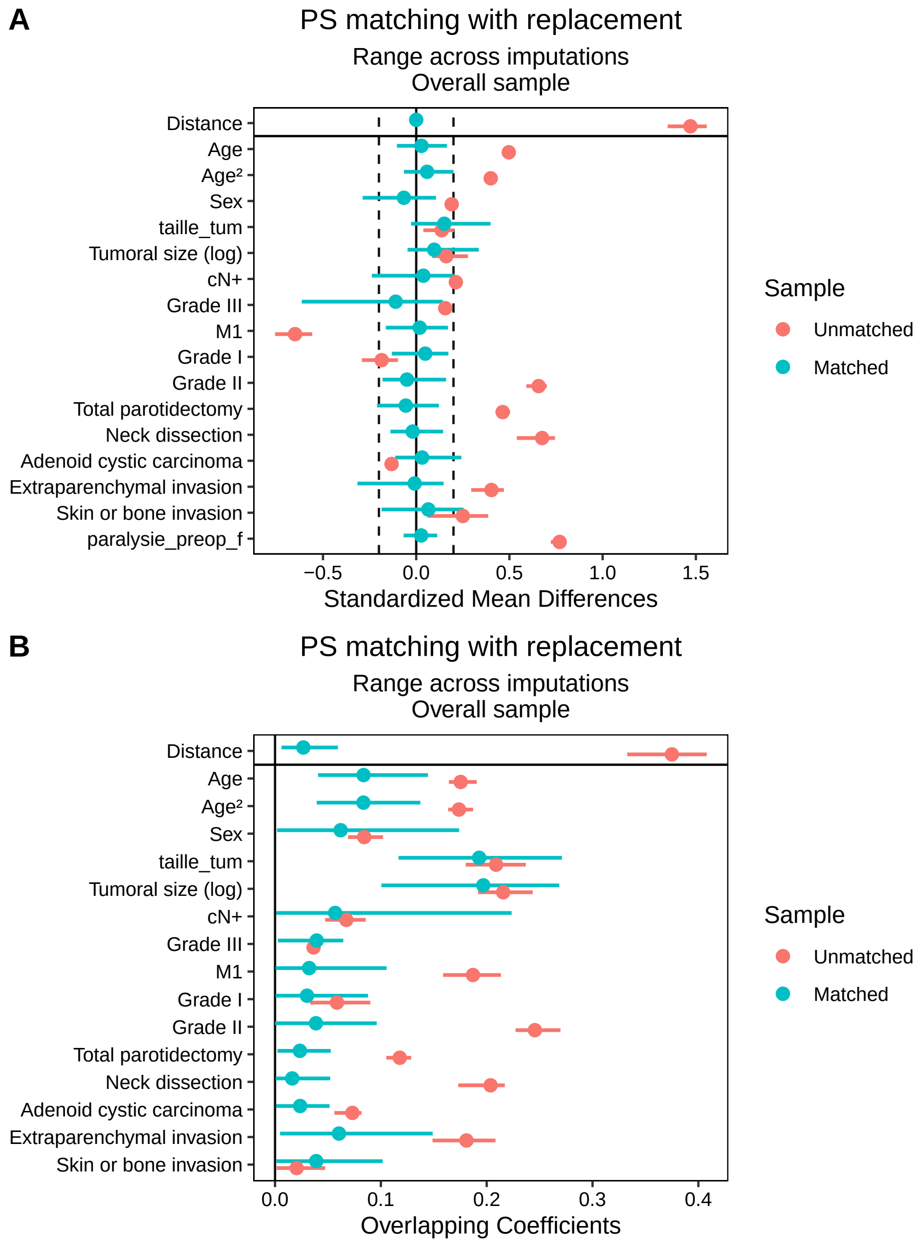

Supplementary Figure 15 Love plot of the balance of variables achieved before and after propensity score matching with replacement. Standardized mean differences (A) and overlapping coefficients (B) for each covariate are presented.

SMRW weighting

The PS model used is $nerve\_resection* \sim age + age^2 + sex + tumoral\_size + log(tumoral\_size) + N + M + grade + total\_parotidectomy + neck\_dissection * +adenoid\_cystic\_carcinoma + extra\_parenchymal\_invasion + skin\_bone\_invasion + pretreatment\_facial\_palsy + pretreatment\_facial\_palsy : grade + grade : log(tumoral\_size) + pretreatment\_facial\_palsy : adenoid\_cystic\_carcinoma + pretreatment\_facial\_palsy : extra\_parenchymal\_invasion$

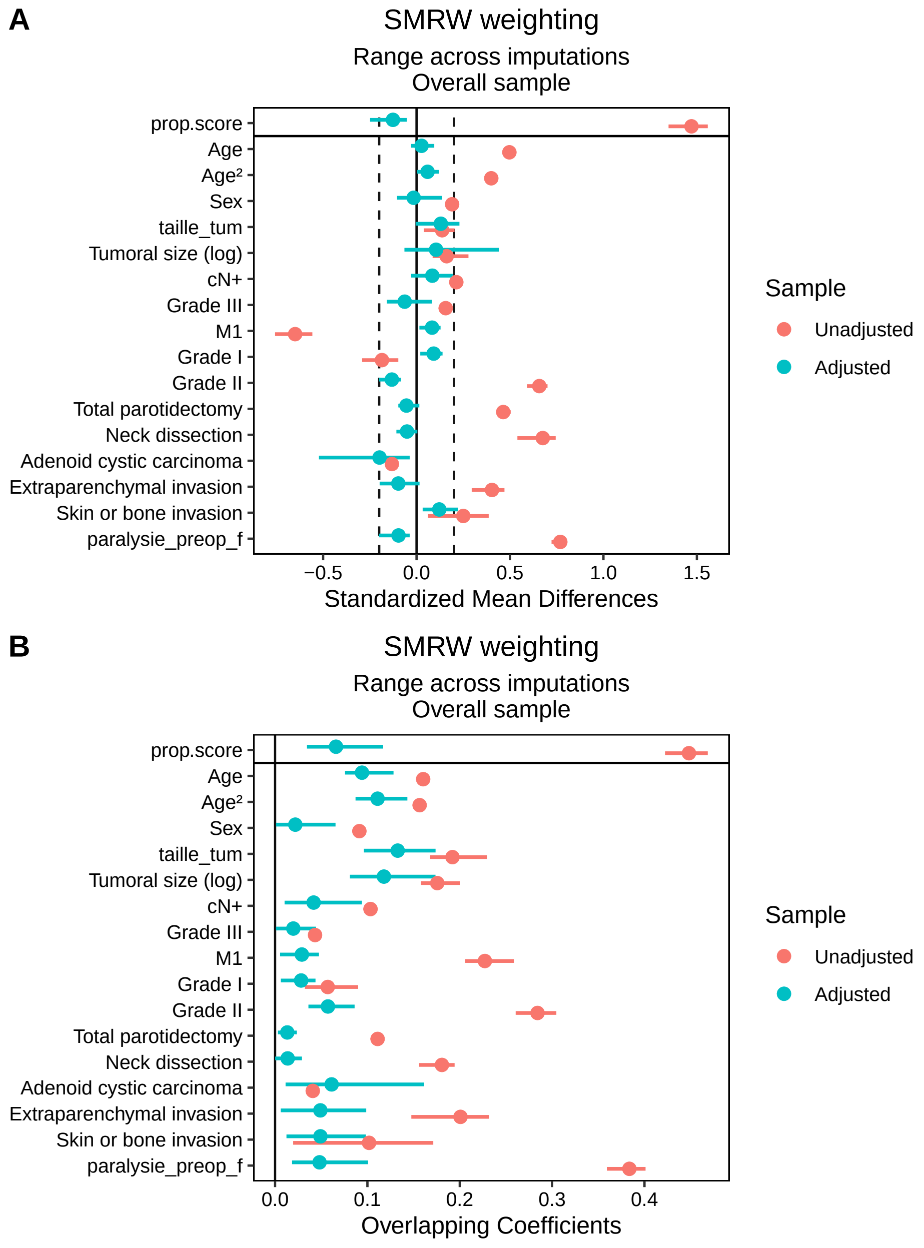

Supplementary Figure 16 Love plot of the balance of variables achieved before and after propensity score weighting with SMRW. Standardized mean differences (A) and overlapping coefficients (B) for each covariate are presented.

Overlap weighting

The PS model used is $nerve\_resection* \sim age + age^2 + sex + tumoral\_size + log(tumoral\_size) + N + M + grade + total\_parotidectomy + neck\_dissection * + adenoid\_cystic\_carcinoma + extra\_parenchymal\_invasion + skin\_bone\_invasion + pretreatment\_facial\_palsy + pretreatment\_facial\_palsy : grade + grade : log(tumoral\_size) + pretreatment\_facial\_palsy : adenoid\_cystic\_carcinoma + pretreatment\_facial\_palsy : extra\_parenchymal\_invasion$

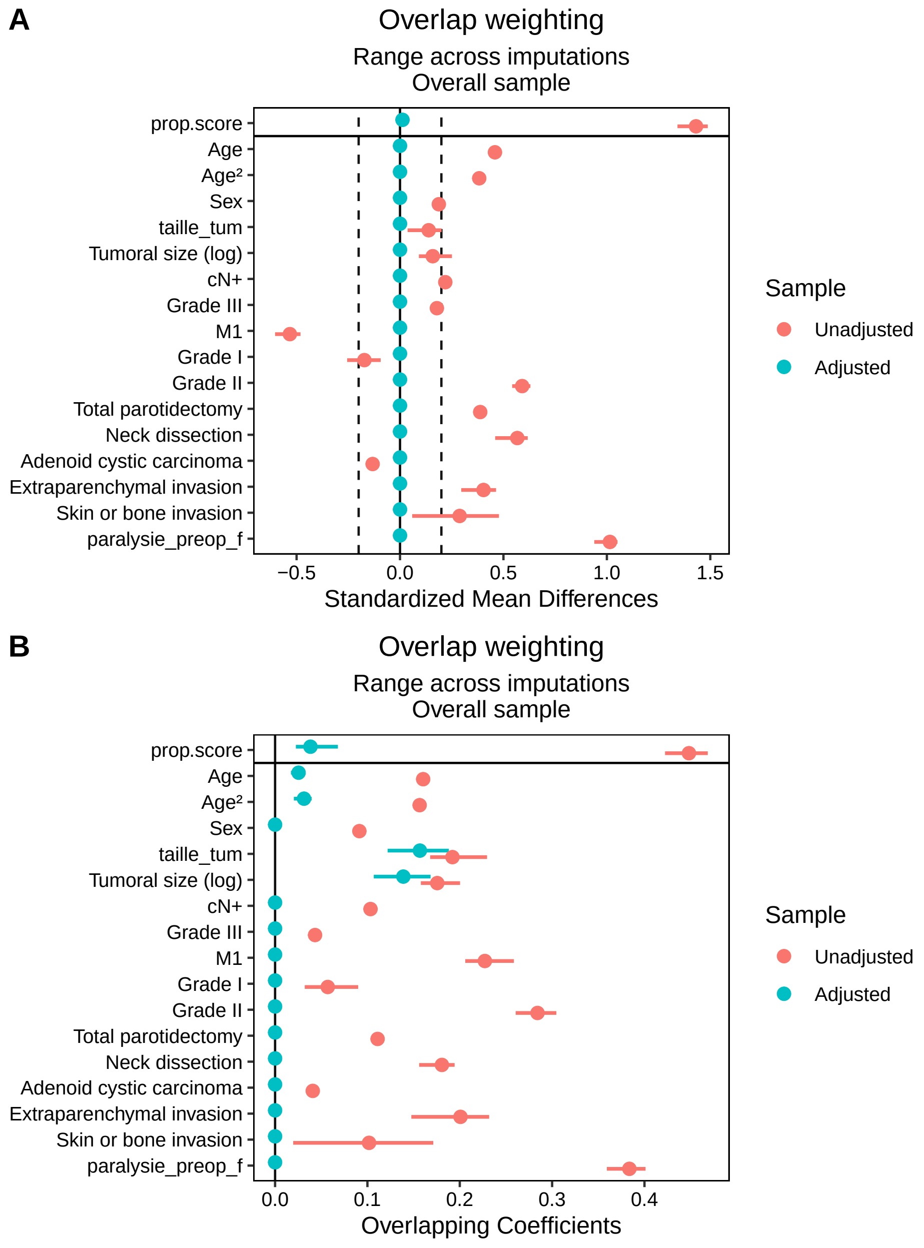

Supplementary Figure 17 Love plot of the balance of variables achieved before and after propensity score weighting with overlap weigths. Standardized mean differences (A) and overlapping coefficients (B) for each covariate are presented.

"Within subsets" strategy

PS matching

The PS model used in the subset with facial palsy is $nerve\_resection* \sim age + sex + tumoral\_size + log(tumoral\_size) + N + M + grade + total\_parotidectomy + neck\_dissection * + adenoid\_cystic\_carcinoma + extra\_parenchymal\_invasion + skin\_bone\_invasion + sex : age + grade : N + grade : log(tumoral\_size) + grade : neck\_dissection* + total\_parotidectomy : skin\_bone\_invasion + total\_parotidectomy : tumoral\_size$

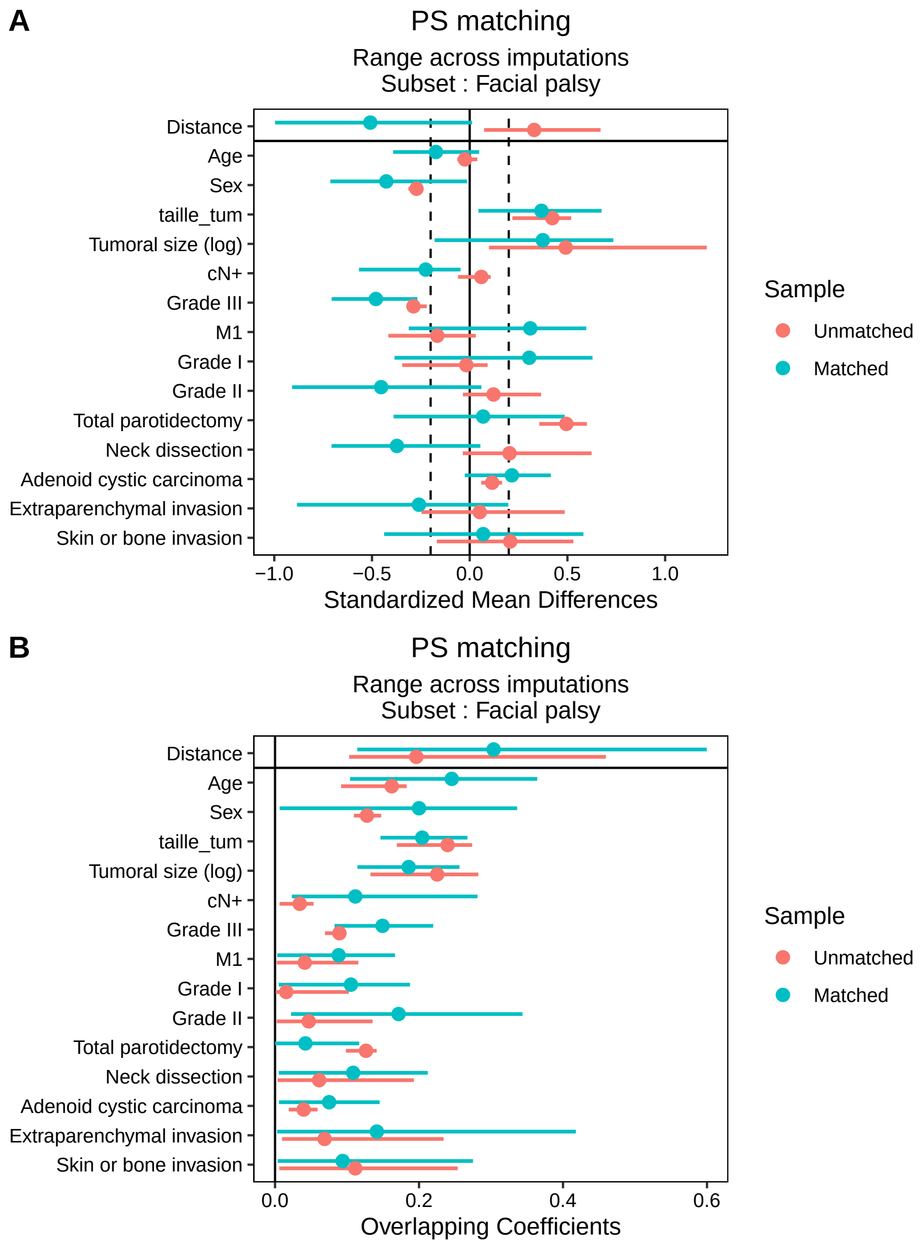

Supplementary Figure 18 Love plot of the balance of variables achieved before and after propensity score matching in the facial palsy subset. Standardized mean differences (A) and overlapping coefficients (B) for each covariate are presented.

The PS model used in the subset without facial palsy is $nerve\_resection* \sim age + age^2 + sex + log(tumoral\_size) + N + M + grade + total\_parotidectomy + neck\_dissection * + adenoid\_cystic\_carcinoma + extra\_parenchymal\_invasion + skin\_bone\_invasion + sex : age + grade : N + grade : log(tumoral\_size) + grade : neck\_dissection * + M : total\_parotidectomy : skin\_bone\_invasion$

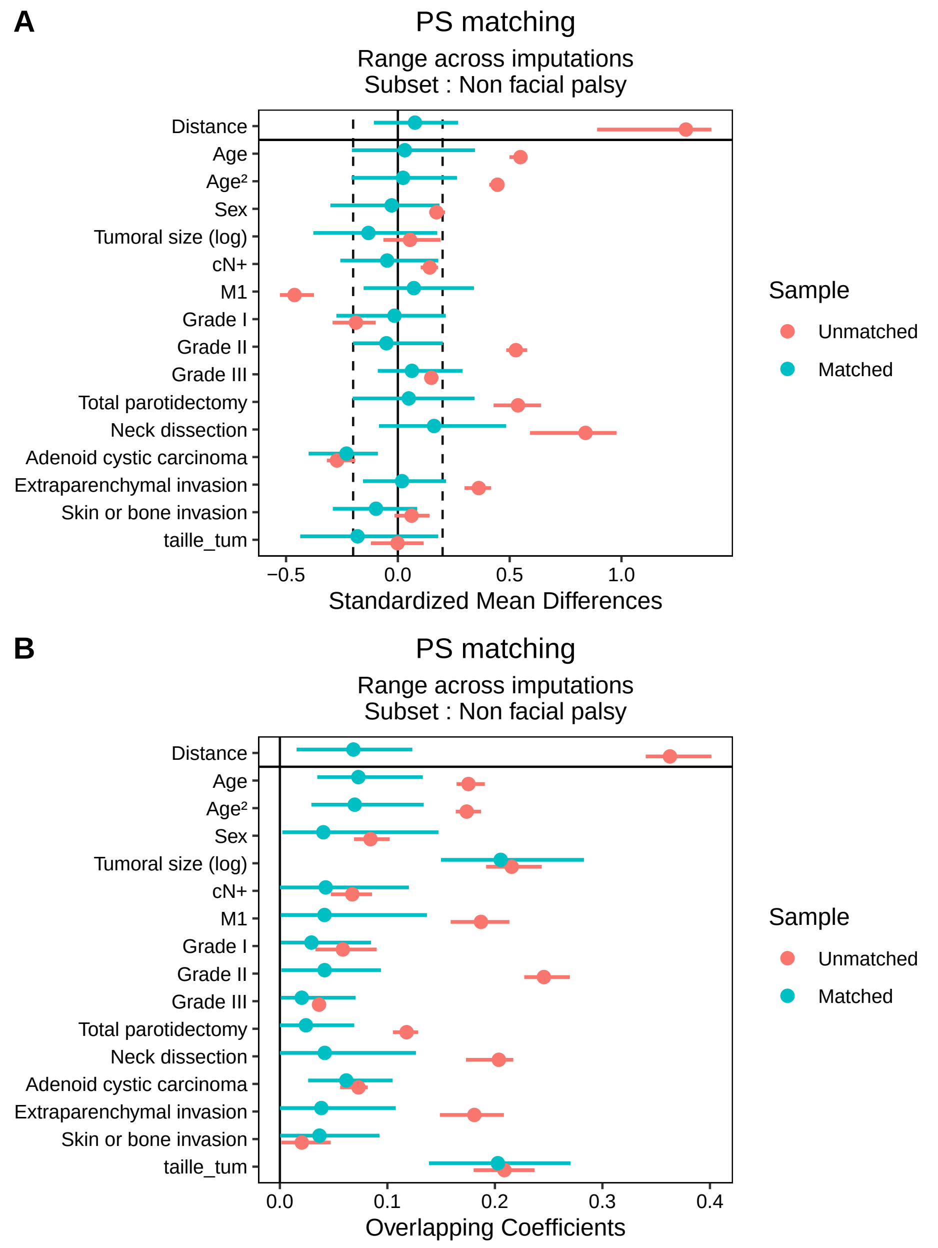

Supplementary Figure 19 Love plot of the balance of variables achieved before and after propensity score matching in the no facial palsy subset. Standardized mean differences (A) and overlapping coefficients (B) for each covariate are presented.

PS matching with replacement

The PS model used in the subset with facial palsy is $nerve\_resection* \sim age + sex + tumoral\_size + log(tumoral\_size) + N + M + grade + total\_parotidectomy + neck\_dissection * + adenoid\_cystic\_carcinoma + extra\_parenchymal\_invasion + skin\_bone\_invasion + sex : age + grade : N + grade : log(tumoral\_size) + grade : neck\_dissection* + total\_parotidectomy : skin\_bone\_invasion + total\_parotidectomy : tumoral\_size + skin\_bone\_invasion : adenoid\_cystic\_carcinoma$

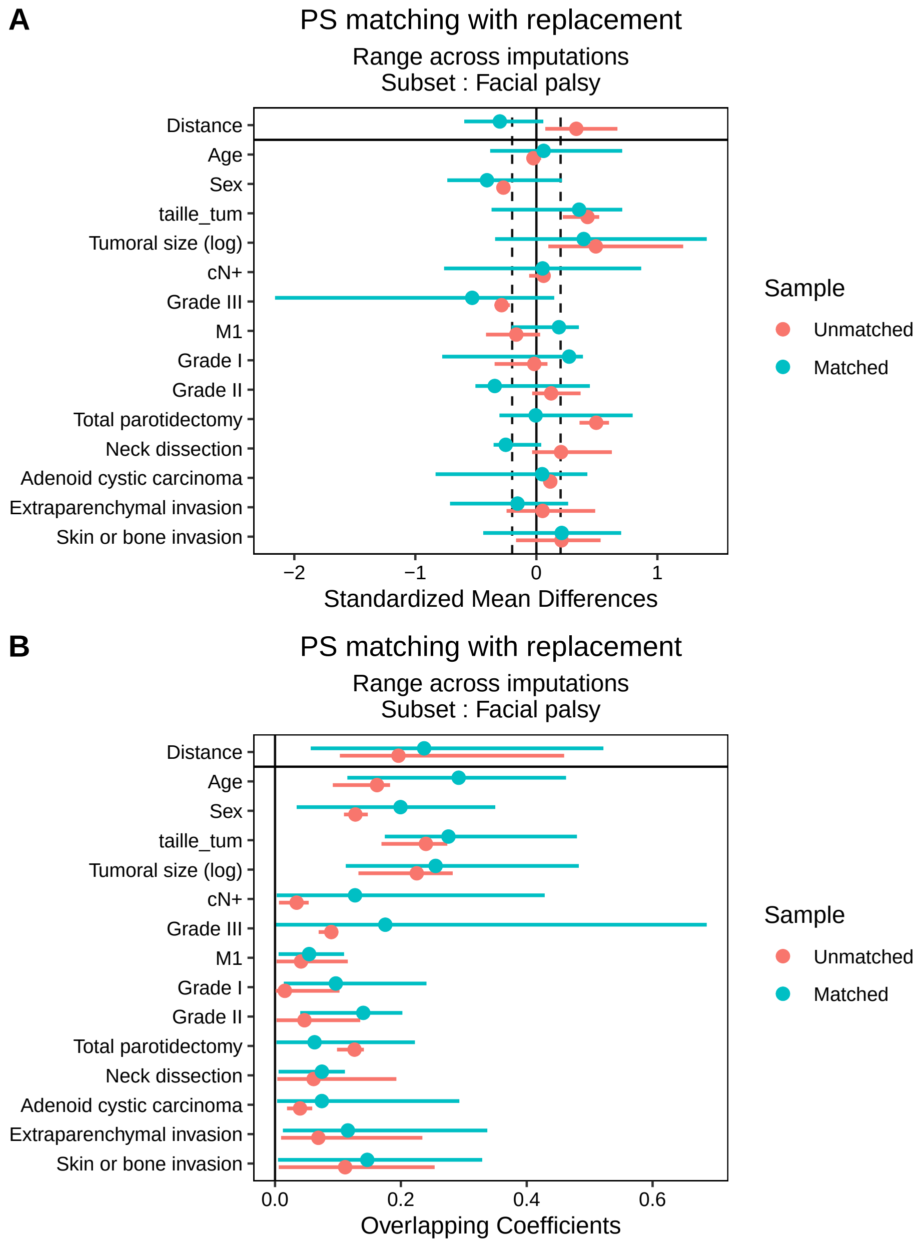

Supplementary Figure 20 Love plot of the balance of variables achieved before and after propensity score matching with replacement in the facial palsy subset. Standardized mean differences (A) and overlapping coefficients (B) for each covariate are presented.

The PS model used in the subset without facial palsy is $nerve\_resection* \sim age + age^2 + sex + log(tumoral\_size) + N + M + grade + total\_parotidectomy + neck\_dissection * + adenoid\_cystic\_carcinoma + extra\_parenchymal\_invasion + skin\_bone\_invasion + grade : N + extra\_parenchymal\_invasion : log(tumoral\_size) + grade : neck\_dissection * + grade : skin\_bone\_invasion + age : skin\_bone\_invasion$

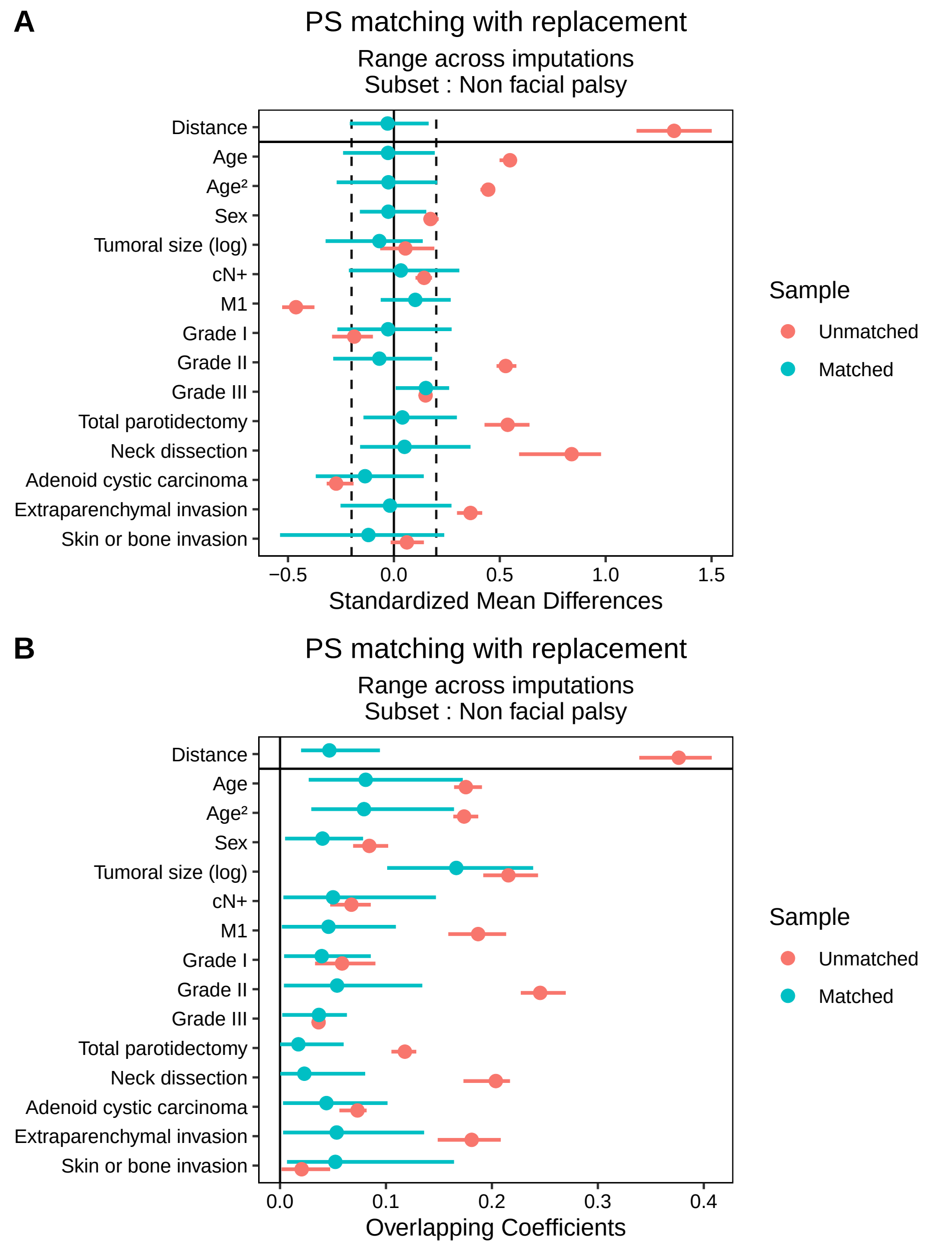

Supplementary Figure 21 Love plot of the balance of variables achieved before and after propensity score matching with replacement in the no facial palsy subset. Standardized mean differences (A) and overlapping coefficients (B) for each covariate are presented.

SMRW weighting

The PS model used in the subset with facial palsy is $nerve\_resection* \sim age + sex + tumoral\_size + log(tumoral\_size) + N + M + grade + total\_parotidectomy + neck\_dissection * + adenoid\_cystic\_carcinoma + extra\_parenchymal\_invasion + skin\_bone\_invasion + sex : age + grade : N + grade : log(tumoral\_size) + grade : neck\_dissection* + total\_parotidectomy : skin\_bone\_invasion + total\_parotidectomy : tumoral\_size$

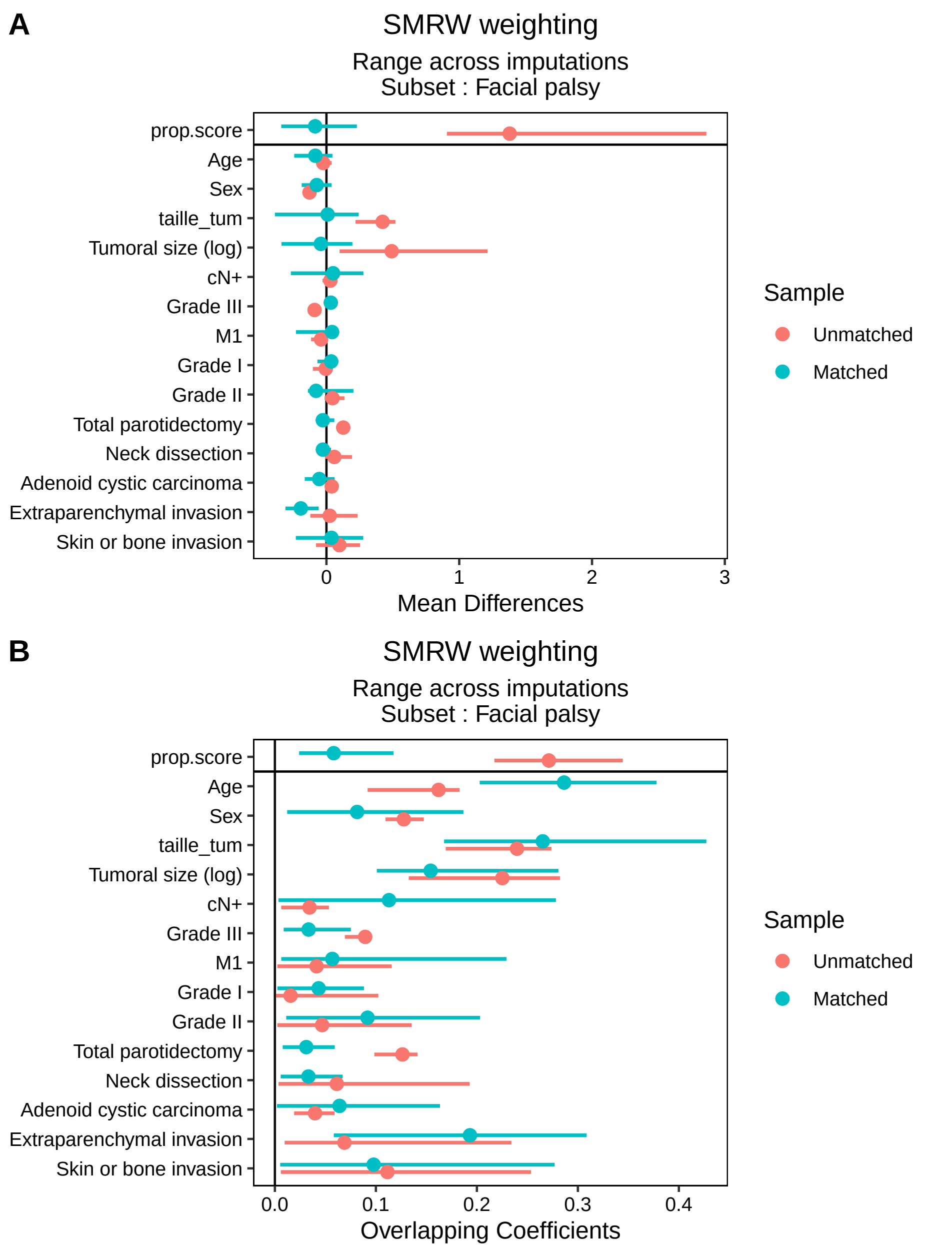

Supplementary Figure 22 Love plot of the balance of variables achieved before and after propensity score weighting with SMRW in the facial palsy subset. Standardized mean differences (A) and overlapping coefficients (B) for each covariate are presented.

The PS model used in the subset without facial palsy is $nerve\_resection* \sim age + age^2 + sex + log(tumoral\_size) + N + M + grade + total\_parotidectomy + neck\_dissection * + adenoid\_cystic\_carcinoma + extra\_parenchymal\_invasion + skin\_bone\_invasion + sex : age + grade : N + grade : log(tumoral\_size) + grade : neck\_dissection * + M : total\_parotidectomy : skin\_bone\_invasion$

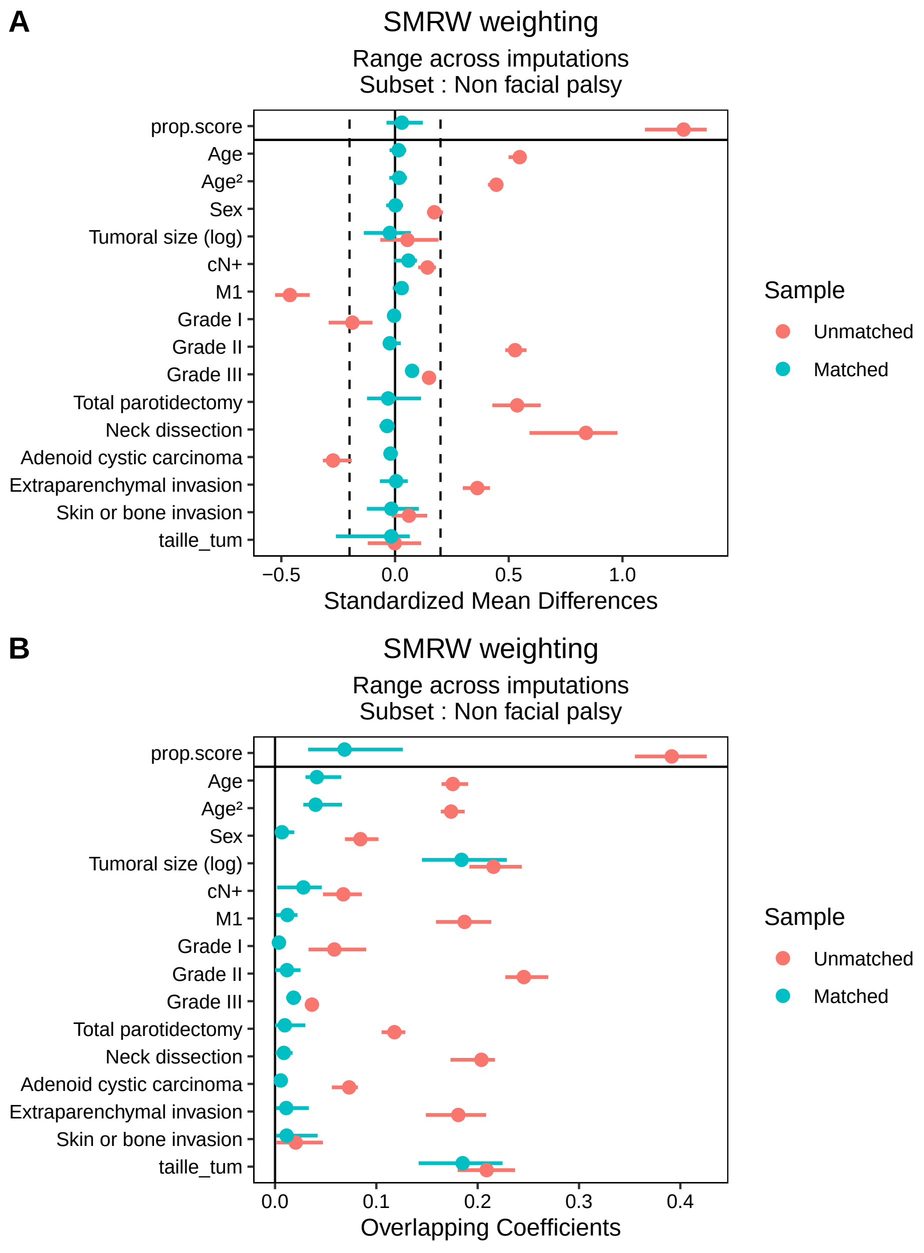

Supplementary Figure 23 Love plot of the balance of variables achieved before and after propensity score weighting with SMRW in the no facial palsy subset. Standardized mean differences (A) and overlapping coefficients (B) for each covariate are presented.

Overlap weighting

The PS model used in the subset with facial palsy is $nerve\_resection* \sim age + sex + tumoral\_size + log(tumoral\_size) + N + M + grade + total\_parotidectomy + neck\_dissection * + adenoid\_cystic\_carcinoma + extra\_parenchymal\_invasion + skin\_bone\_invasion + sex : age + grade : N + grade : log(tumoral\_size) + grade : neck\_dissection* + total\_parotidectomy : skin\_bone\_invasion + total\_parotidectomy : tumoral\_size$

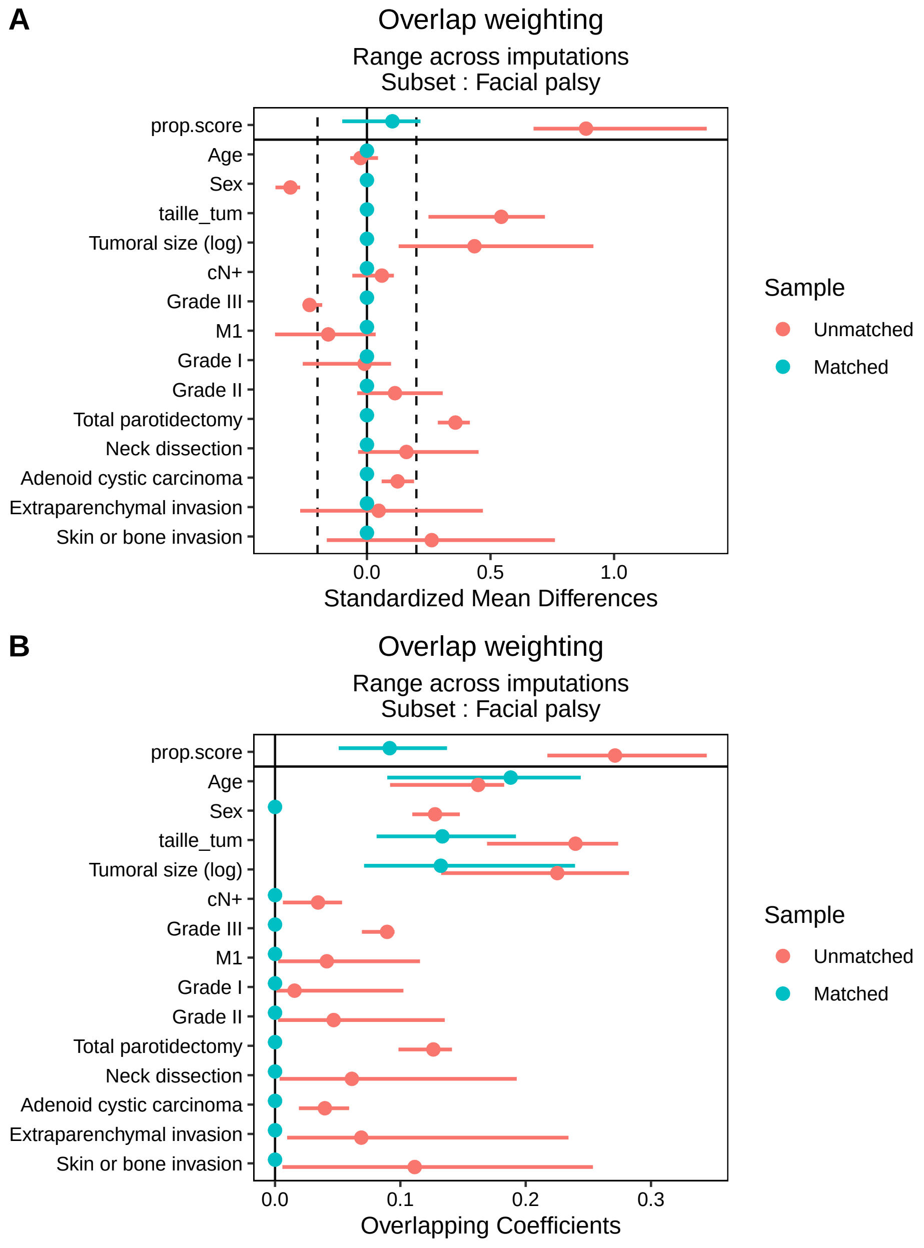

Supplementary Figure 24 Love plot of the balance of variables achieved before and after propensity score weighting with overlap weights in the facial palsy subset. Standardized mean differences (A) and overlapping coefficients (B) for each covariate are presented.

The PS model used in the subset without facial palsy is $nerve\_resection* \sim age + age^2 + sex + log(tumoral\_size) + N + M + grade + total\_parotidectomy + neck\_dissection * + adenoid\_cystic\_carcinoma + extra\_parenchymal\_invasion + skin\_bone\_invasion + sex : age + grade : N + grade : log(tumoral\_size) + grade : neck\_dissection * + M : total\_parotidectomy : skin\_bone\_invasion$

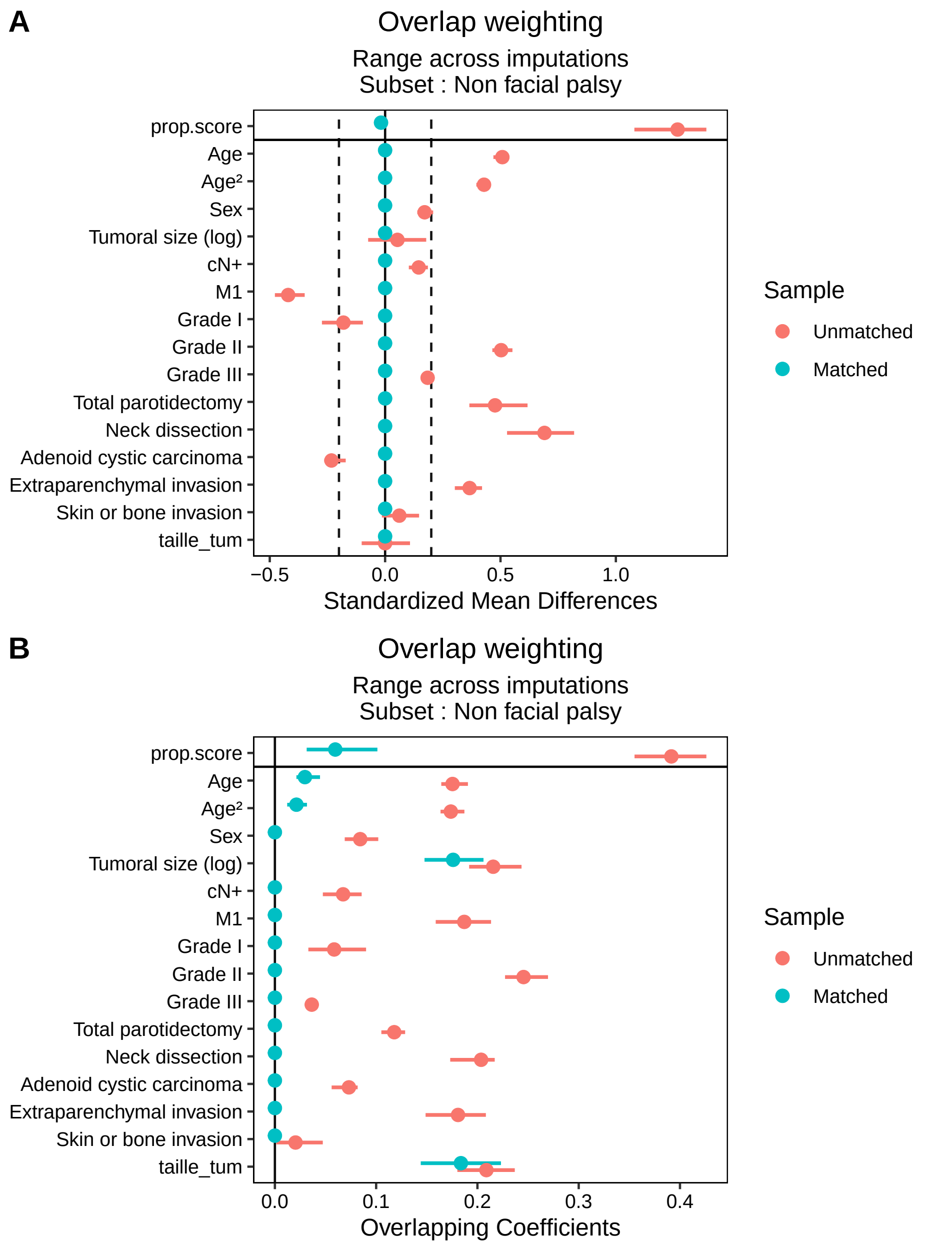

Supplementary Figure 25 Love plot of the balance of variables achieved before and after propensity score weighting with overlap weights in the no facial palsy subset. Standardized mean differences (A) and overlapping coefficients (B) for each covariate are presented.
